# Kölliker's Organ Functions as a Developmental Hub in Mouse Cochlea Regulating Spiral Limbus and Tectorial Membrane Development

**DOI:** 10.1523/JNEUROSCI.0721-24.2025

**Published:** 2025-02-05

**Authors:** Hongji Zhang, Timothy Papiernik, Selena Tian, Amal Yaghmour, Ahmad Alzein, James Benjamin Lennon, Rahul Maini, Xiaodong Tan, Ava Niazi, Joosang Park, Sungjin Park, Claus-Peter Richter, Michael Ebeid

**Affiliations:** ^1^Anatomy Department, Midwestern University, Downers Grove, Illinois 60515; ^2^College of Graduate Studies, Midwestern University, Downers Grove, Illinois 60515; ^3^Chicago College of Osteopathic Medicine, Midwestern University, Downers Grove, Illinois 60515; ^4^Department of Otolaryngology, Feinberg School of Medicine Northwestern University, Chicago, Illinois 60611; ^5^The Hugh Knowles Center, Department of Communication Sciences and Disorders, Northwestern University, Evanston, Illinois 60208; ^6^Neuroscience Program, University of Utah, Salt Lake City, Utah 84112; ^7^Department of Neurobiology, University of Utah, Salt Lake City, Utah 84112; ^8^Departments of Biomedical Engineering, Northwestern University, Evanston, Illinois 60208; ^9^Communication Sciences and Disorders, Northwestern University, Evanston, Illinois 60208

**Keywords:** cochlea, development, Kölliker's organ, *Prdm16*, spiral limbus, tectorial membrane

## Abstract

Kölliker's organ is a transient developmental structure in the mouse cochlea that undergoes significant remodeling postnatally. Utilizing an epithelial-specific conditional deletion mouse model of *Prdm16* (marker and regulator of Kölliker's organ), we show that *Prdm16* is required for interdental cell development, and thereby the development of the limbal domain of the tectorial membrane and its medial anchorage to the spiral limbus. Additionally, we show that Kölliker's organ is involved in normal tectorial membrane collagen fibril development and maturation. Interestingly, mesenchymal cells of the spiral limbus underneath *Prdm16*-deficient Kölliker's organ failed to produce interstitial matrix proteins, resulting in a hypoplastic and truncated spiral limbus, indicating a non-cell autonomous role of *Prdm16* in regulating spiral mesenchymal matrix development. Single-cell RNA sequencing identified differentially expressed genes in *Prdm16*-deficient Kölliker's organ suggesting a role for connective tissue growth factor (CTGF) downstream *Prdm16* in epithelial-mesenchymal signaling involved in spiral limbus matrix deposition. *Prdm16*-deficient mice showed a hearing deficit, as indicated by elevated auditory brainstem response thresholds at most frequencies, consistent with the cochlear structural defects. Both sexes were studied. This work establishes *Prdm16* as a deafness gene in mice through its role in regulating Kölliker's organ development. Such understanding recognizes Kölliker's organ as a developmental hub regulating multiple surrounding cochlear structures.

## Significance Statement

In this study, we show that the Kölliker's organ functions as a developmental hub that orchestrates the development of tectorial membrane and spiral limbus during cochlear development. Utilizing a mouse model of conditional deletion of *Prdm16* (marker and regulator of Kölliker's organ), we establish *Prdm16's* necessity for hearing in mice through its many roles during cochlear development including permitting interdental cell development and thereby the formation of the tectorial membrane limbal domain, secreting collagens essential for tectorial membrane matrix development, and signaling to the underlying mesenchyme to secrete extracellular matrix and develop the spiral limbus.

## Introduction

Mammalian inner ear consists of the cochlea, which functions in sound perception, and the vestibular system which is involved in the sense of balance and motion. The mammalian cochlea develops as a ventral outgrowth from a fluid-filled cyst (otocyst) that is a derivative of the ectoderm adjacent to the developing hindbrain. As the cochlear duct develops, its floor becomes subdivided into three domains according to gene expression signatures: the medial (modiolar) Kölliker's organ domain (also known as greater epithelial ridge; GER) expressing *Fgf10* and *Prdm16* (Pauley et al., 2003; Ebeid et al., 2022) that gives rise to the inner sulcus, a middle prosensory domain, expressing *Sox2* and *Lfng* that develops into the organ of Corti (Zhang et al., 2000; Hume et al., 2007), and a lateral lesser epithelial ridge domain expressing *Bmp4* that develops into the outer sulcus (Morsli et al., 1998). During the early postnatal period, Kölliker's organ undergoes significant remodeling, likely due to programmed cell death (Knipper et al., 1999; Kamiya et al., 2001; Takahashi et al., 2001), giving rise to the cells of the inner sulcus (Hinojosa, 1977). The role of Kölliker's organ during development and before remodeling is still under investigation and is thought to be involved in generating spontaneous electric activity in the developing organ of Corti (Tritsch et al., 2007; Tritsch and Bergles, 2010) and in the secretion of tectorial membrane components including collagens and glycoproteins such as otogelin and tectorin (Anniko, 1980; Lim and Anniko, 1985; Lim, 1987; Rau et al., 1999; El-Amraoui et al., 2001).

The tectorial membrane is an apical extracellular matrix structure essential for coupling sound-induced vibrations to the hair cell stereocilia. It spirals along the length of the organ of Corti and is attached along its medial edge to the spiral limbus, via a single row of radially ordered epithelial cells (the interdental cells). Abnormalities of the tectorial membrane structure have been linked to sensorineural hearing loss, primarily due to deletion in genes encoding tectorin proteins (Verhoeven et al., 1998; Xia et al., 2010; Legan et al., 2014). During development, the spiral limbus originates as an outgrowth from the mesenchyme underlying the Kölliker's organ and interdental cells (Thorn et al., 1979). Mechanisms regulating the spiral limbus mesenchymal cell development and maturation are not clear.

Our recent work has identified the expression of PR domain-containing 16 (PRDM16) in the developing Kölliker's organ of mouse cochleae (Ebeid et al., 2022). This protein is a member of the PRDM family that functions as a transcriptional regulator in diverse cell types, including adipose, neural, cardiac, and hematopoietic tissues (Seale et al., 2007; Bjork et al., 2010; Chuikov et al., 2010; Aguilo et al., 2011; Warner et al., 2013). *Prdm16^cGT^* null cochlea exhibited hypoplastic Kölliker's organ, lack of spiral limbus, and deformed tectorial membrane at birth (Ebeid et al., 2022). Since *Prdm16^cGT^* null mice die around postnatal day (P) 0, this work aimed to characterize a cochlear-specific *Prdm16* conditional knockout mouse model as a tool to uncover Kölliker's organ roles before remodeling and hearing onset.

This study provides the first evidence, to our knowledge, of the necessity of *Prdm16* for hearing function via its regulation of tectorial membrane, spiral limbus, and interdental cell development. The data from this study will help clinicians understand the pathophysiology of hearing loss in 1p36 deletion syndrome (Monosomy 1p36), one of the most common chromosome deletion syndromes that occurs due to a heterozygous deletion of the distal part of the short arm of chromosome 1 that includes *Prdm16* gene (Slavotinek et al., 1999; Shaffer and Heilstedt, 2001; Heilstedt et al., 2003).

## Materials and Methods

### Ethics statement

This study followed the guidelines in the Guide for the Care and Use of Laboratory Animals of the National Institutes of Health. The Midwestern University Institutional Animal Care and Use Committee approved the protocol (Protocol# 3130). All efforts were made to minimize animal suffering.

### Animals

The *Prdm16^fl/fl^* mouse strain (JAX 024992) possessing loxP sites flanking exon 9 of the *Prdm16* gene was used. *Prdm16^fl/fl^* female mice were crossed with *Fgf20^Cre/Cre^* male mice (Huh et al., 2015) to generate *Prdm16^fl/+^*; *Fgf20^Cre/+^* male mice which were then mated with *Prdm16^fl/fl^* female mice to generate *Prdm16* conditional knock-out (cKO; *Fgf20^Cre/+^*; *Prdm16^fl/fl^*) and heterozygote littermate control (*Fgf20^Cre/+^*; *Prdm16^fl/+^*) mice. The *Prdm16^fl/fl^* strain was maintained on a C57BL/6J inbred strain background (Jax: 000664). To investigate *Fgf20^Cre^* activity during development, *Rosa^td-Tomato^* (Ai14) Cre reporter strain (Jax: 007914) was used that express td-Tomato following the Cre-mediated removal of a loxP-flanked STOP cassette, thus marking Cre activity domain and lineage (Madisen et al., 2010). The *Prdm16^cGT^* mutant mouse strain was previously reported (Strassman et al., 2017). *Prdm16^cGT/+^* male and Wild-type (WT) female mice were mated to generate heterozygote (*Prdm16^cGT/+^*) and WT (*Prdm16^+/+^*) littermate controls. The *Prdm16^cGT/+^* mouse strain was maintained on an FVB/NJ inbred strain background (Jax: 001800). For all experiments, both sexes were studied. Time-mated females were checked daily for presence of post copulatory vaginal plugs, and if present, the developmental stage of the litter was considered at embryonic day (E) 0.5.

### Immunostaining

P0 pups were decapitated after anesthesia using hypothermia for 2–4 min on crushed ice. P7, P14, and P21 mice were transcardially perfused with 4% paraformaldehyde under anesthesia with an intraperitoneal injection of a mixture of ketamine hydrochloride (100 mg/kg) and xylazine hydrochloride (10 mg/kg). The temporal bones (including the inner ears) were dissected, fixed with 4% paraformaldehyde overnight at 4°C, washed with phosphate-buffered saline (PBS) three times, and then decalcified using 10% ethylenediaminetetraacetic acid (EDTA) at 4°C until the tissues softened. For cryosection, samples were emersed in ascending sucrose concentrations (10, 20, and 30%) for 1 h, then in 1:1 30% sucrose and OCT overnight, then embedded in OCT, and frozen on dry ice. Paraffin processing was done using Sakura Tissue-Tek VIP 5 Tissue Processor as follows: dehydration in ascending ethanol concentrations (50, 70, 80, 95, and 100%) for 1 h each, followed by two 100% ethanol washes, three Microclear washes (1 h each), and two paraffin washes (1 h each) followed by paraffin embedding using Sakura Tissue-Tek TEC Paraffin Embedding Station. Embedded samples were serially sectioned horizontally (10 µm thick for cryosections and 6 µm for paraffin sections) using a cryostat (Leica CM1950) and a Thermo Scientific Microm HM325 Rotary Microtome, respectively. Paraffin sections were floated on 45°C water bath, mounted onto slides, air-dried at 40°C, washed three times in xylene and descending grades (100, 95, 70%) of ethanol for 5 min each, and then were placed through antigen retrieval by boiling slides in citrate buffer, pH 6, for 8 min at 95°C. Paraffin or cryosections were permeabilized with PBS containing 0.1% Tween 20 and then blocked with PBS containing 0.1% Tween 20 and 5% normal donkey serum (Southern Biotech 0030-01) and incubated in primary antibody overnight at 4°C. Sections were then washed with PBS, incubated with a secondary antibody for 2 h at room temperature, washed, mounted in DAPI mounting media, and imaged with a Nikon A1R confocal microscope. For whole-mount immunostaining, the basilar membrane along with the overlying cochlear epithelium were microdissected, permeabilized with PBS containing 0.5% Triton, blocked with PBS containing 0.5% Triton and 5% normal donkey serum, and incubated in primary antibody overnight at 4°C. Samples were washed with PBS and incubated with a secondary antibody for 2 h at room temperature, washed, placed on a glass microscope slide in DAPI mounting media, coverslipped, and photographed using a Nikon A1R confocal microscope. Endogenous tissue background control (no antibodies added) and a nonprimary antibody control (no primary antibody with secondary antibody added) were used as controls for immunostaining. Primary antibodies/stains used: phalloidin (R&D Systems, 1:100), MYO6 (Proteus, 1:200), SOX2 (R&D Systems, 1:200), PRDM16 (Strassman et al., 2017), COLLAGEN II (Abcam, 1:200), COLLAGEN IV (Millipore Sigma, 1:200), COLLAGEN IX (DHSB, 1:50), COCHLIN (Sigma-Aldrich, 1:200), TGFBI (Abcam, 1:200), VIMENTIN (Cell Signaling, 1:200), SOX9 (MilliporeSigma, 1:200), and Activated CASPASE 3 (Cell Signaling, 1:200). Concentrations are determined based on manufacturer's recommendation or previous publications (Strassman et al., 2017).

### Hematoxylin and eosin and trichrome staining

Paraffin sections were stained with Mayer's hematoxylin (Sigma-Aldrich MHS16) and eosin (Sigma-Aldrich HT110216) or Trichrome Stain Kit (Abcam, ab150686) according to manufacturer's protocol (chrome-extension://efaidnbmnnnibpcajpcglclefindmkaj/https://www.abcam.com/ps/products/150/ab150686/documents/Trichrome-Stain-Kit-protocol-book-v3d-ab150686%20(website).pdf). Images were captured using Nikon Eclipse Ti2-E Inverted Microscope equipped with color camera.

### Scanning electron microscopy and transmission electron microscopy

Mice were anesthetized using intraperitoneal injection of a mixture of ketamine hydrochloride (100 mg/kg) and xylazine hydrochloride (10 mg/kg) and then transcardially perfused with 2.5% glutaraldehyde. The cochleae were dissected, then a small hole was made in the apical cochlear capsule along with opening of the oval window, and 20 µl of 2.5% glutaraldehyde with 1% tannic acid in 0.1 M sodium cacodylate buffer, pH 7.2, was perfused through the oval window and the apical hole. Cochleae were immersed in the same fixative at 4°C for 24 h. After three washes in 0.1 M sodium cacodylate buffer at pH 7.2, cochleae were then fixed with 1% osmium tetroxide in 0.1 M sodium cacodylate for 10 min at room temperature, followed by three washes in 0.1 M sodium cacodylate buffer and decalcification in 10% EDTA, pH 8.0, for 7 d. For SEM, the basilar membrane was dissected, dehydrated in ascending grade of ethanol (25, 50, 75, 95, 100%), and kept overnight in 100% ethanol at 4°C. A critical point dryer (Leica EM CPD300) was used to dry the samples, which were then mounted on disks and sputter coated with 10 nm sliver using Leica EM ACE600 High Vacuum Coater. Samples were then imaged by Jeol scanning electron microscope at different magnifications. For TEM, cochleae were dehydrated through ascending ethanol concentrations, infiltrated, and embedded with resin (TAAB 812 resin kit, TAAB Laboratories, E202/1). In brief, the cochleae were sequentially immersed in a mixture of resin and acetone, starting with a 50:50% (v/v) ratio for 2 h, 70:30% (v/v) overnight, 100% resin for 8 h, and another 100% resin incubation overnight. The specimens were placed in embedding capsules (10504, Ted Pella) and cured at 60°C for 72 h. The samples were radially sliced to a thickness of 1 μm using the Leica UC6 Ultramicrotome and further sectioned into a thickness ranging from 40 to 100 nm using a diamond knife on the Leica UC6 Ultramicrotome at the University of Utah's Electron Microscopy Core Laboratory. The resulting sections were affixed onto copper grids (200 mesh), subsequently stained in sequence with saturated aqueous uranyl acetate followed by Reynold's Lead Citrate, and imaged on JEOL JEM-1400 transmission electron microscopes at the University of Utah Electron Microscopy Core Laboratory.

### RNA fluorescence in situ hybridization

Cryosections (10 μm) were used for RNA FISH analysis following the manufacturer's protocol (Molecular Instruments, HCR RNA-FISH protocol for fresh frozen or fixed frozen tissue sections https://files.molecularinstruments.com/MI-Protocol-RNAFISH-FrozenTissue-Rev2.pdf). Custom HCR probe sets were designed as multiple probe pairs that hybridize to different sequences along the target mRNA (Molecular Instruments).

### Single-cell (Sc) RNA sequencing and analysis

E16.5 embryos carrying *Prdm16* conditional deletion and heterozygote controls (*n* = 3/group) were collected, and the cochleae were microdissected in sterile cold Dulbecco's Modified Eagle Medium (DMEM), then incubated in 0.25% trypsin-EDTA (Invitrogen) for 15 min at 37°C with gentle trituration every 5 min, followed by trypsin inactivation (adding an equal volume of DMEM). Dissociated cells were passed through a 30 μm strainer (Fisherbrand), pelleted at 300 × g for 5 min and then resuspended in 100 μl of cold PBS supplemented with 1% fetal bovine serum (Invitrogen). Single cells were captured and lysed, and mRNAs were reverse transcribed into cDNAs using a 10x Genomics Chromium Controller at Northwestern University Sequencing core. cDNA libraries were prepared using Chromium Single Cell 3′ according to the manufacturer's instructions. Libraries were sequenced on a NovaSeq S2 PE50 Sequencer. 10x Genomics Cloud Analysis was used to align sequences to the Ensemble mouse MM10 assembly using Cell Ranger 2.1.1 analysis software (10× Genomics). Processing of the Cell Ranger output data was done with Loupe Browser v.8 (10× Genomics). Gene expression-based clustering information including t-SNE projections and differential gene expression were done utilizing Loupe Browser v.8. Differential gene expression was done using a pseudobulk calculation. For this calculation, Loupe Browser's algorithm creates a new matrix for each cluster and then runs sSeq to identify differential genes between experimental and control samples (Yu et al., 2013). Sequencing data is deposited in the gEAR database and can be accessed through the following link: https://umgear.org/p?l=33372cd0.

### Auditory brainstem responses

Animals were anesthetized with a mixture of Ketamine (80–100 mg/kg)/Xylazine (2–10 mg)/kg) given intraperitoneally. The body temperature was maintained with a heating pad at 37°C. Auditory brainstem response (ABR) recordings were performed with the Tucker-Davis Technologies (TDT) system (RZ6 processor and BioSigRZ software) at Midwestern University. After the mouse was anesthetized, three electrodes were placed subdermally behind the tested ear (channel 1), the vertex (reference), and behind the opposite ear (ground). MF-1 magnetic speaker (Tucker-Davis Technologies) was placed 5 cm in front of the right ear. Click and tone signals were calibrated using a 0.25 inch Free-Field Cartridge Microphone (PCB 377C01). For click stimulus, a 0.1 ms, single-channel mono-phasic click was presented at a rate of 21/second. With each successive presentation, the click phase alternated between a condensation and rarefaction click to eliminate potential speaker artifacts. Click level varied from 90 to 10 dB SPL in 10 dB decrements. For tone stimuli, a 5 ms, single-channel cosine-squared gated tone (2 ms rise/fall) was presented 21 times per second at the following frequencies: 4, 8, 16, and 32 kHz. For each frequency, the tone level varied from 90 to 20 dB SPL in 10 dB decrements. With each successive presentation, the tone phase alternated 180° to eliminate potential speaker artifacts. For both click and tone stimuli, a 10 ms response window was acquired from a single acquisition channel on the preamplifier (MEDUSA4Z, TDT) via the RZ6 fiber optic port. A total of 512 responses were averaged at each frequency and level combination. The recordings were bandpass filtered (300–3,000 Hz) and averaged over 512 repetitions. A notch filter (60 Hz) was used. Hearing thresholds are the minimum dB SPL that elicits a wave above the noise floor. Peak I amplitude was measured from the first peak to the trough that immediately follows peak I.

### Synchrotron x-ray microcomputed tomography

Inner ears were fixed in 4% PFA in PBS overnight, washed, and then placed in 10% ethylene glycol tetraacetic acid (EGTA) in PBS overnight for partial decalcification. Microcomputed tomography (micro-CT) was performed using synchrotron radiation at the 2-BM beamline at the Advanced Photon Source at Argonne National Laboratory in Argonne, Illinois. A 25.5 keV monochromatic beam was used. Images were acquired using a 20 μm LuAG scintillator and a CCD-based detector fit with a 2× objective lens. The resolution was 1.7 μm. The field of view was 8 × 1.8 mm. The sample-to-detector distance was 600 mm. Projections were acquired over 180° of rotation in fly scan mode (continuous sample rotation). Flat- and dark-field images were captured to correct irregularities in the x-ray beam or the detector during the tomographic reconstruction. Flat-field images are captured when the x-ray beam is on, but the sample is out of the field of view. Dark-field images are captured when a shutter blocks the x-ray beam. Tomographic reconstructions were completed with custom code using the open-source TomoPy Python package. Reconstructed stacks had 1,080 32 bit grayscale images with isotropic voxel size.

Slice images were visualized and analyzed using Amira software (Thermo Fisher Scientific). The scala media, spiral limbus, and tectorial membrane were manually outlined and segmented. A conversion factor of 1.7 µm per pixel was used to convert pixel values to micrometer. Surface generation was used to generate three-dimensional models, and volume rendering for each structure was performed in Amira according to the manual (https://assets.thermofisher.com/TFS-Assets/MSD/Product-Guides/users-guide-amira-software-2019.pdf). 3D slicer software was utilized to compute 3D model measurements (Fedorov et al., 2012). Scala media, tectorial membrane, and spiral limbus lengths were determined by creating a sequence of 100 coordinate points along the length of each structure where each point bisects a perpendicular line joining the medial and the lateral border of the structure, then creating a line joining the 100 coordinate points and measuring its length.

### Cochlear duct RNA isolation and quantitative real-time PCR

Inner ears were dissected and incubated in Dispase (1 U/ml) in DMEM/F-12 (Stemcell Technologies) for 15 min at 37°C. Then, the otic capsule was opened, and the cochlear duct was dissected from the surrounding mesenchymal tissues and placed in RNA extraction buffer and then homogenized (Bel-Art 650000000). RNA extraction and subsequent removal of genomic DNA were performed using RNAqueous RNA isolation kit (Invitrogen) according to the manufacturer's protocol. Reverse transcription of total RNA was performed using GoScript Reverse Transcription System (Promega) according to the manufacturer’s protocol. Quantitative real-time PCR (qPCR) was performed using TaqMan Fast Advanced Master Mix (Thermo Fisher Scientific) according to manufacturer's protocols. The TaqMan primers used were as follows: *Gapdh* (4453320), *Ctgf* (4453320), and *Calb1* (4331182). Data analysis was performed using the comparative CT method (Schmittgen and Livak, 2008), and data was normalized to detection of *Gapdh* mRNA.

### Ex vivo embryonic cochlear culture and electroporation

E14.5 embryos from WT C57BL/6 pregnant mice were dissected, and the cochleae were electroporated with either *Prdm16* overexpressing plasmid (Addgene pcDNA3.1 PRDM16; 15503) or control backbone plasmid (Addgene pcDNA3.1; 172604) as previously described (Driver and Kelley, 2010). Electroporated cochleae were plated on micelle membranes and maintained in vitro for 4 d as previously described (Ogier et al., 2019). Tissues were fixed with 4%PFA for 15 min at room temperature, then immunoassayed, and imaged as previously described in this study.

### Data analysis and statistics

Data analysis of immunostained samples was done using ImageJ (version 1.54f; Schindelin et al., 2012). The cochlear sensory epithelium length was measured from the tip of the apex to the base using MYO6 immunostaining. HC and SC densities were determined by counting the number of MYO6+ and SOX2+ cells, respectively, within the sensory epithelium along the length of the cochlear duct and normalized to 100 µm. Base, middle, and apical refer to the basal one third, middle one third, and apical one third of the cochlear epithelium. We used consistent laser power, gain, and pinhole size to quantify fluorescence intensity in control and cKO-stained sections. Using Fiji, we merged 10 optical slices (0.5 µm each), selected the area of interest, and measured the area, mean intensity, integrated density, and three different background mean intensities from the same slide with the same area. Corrected mean fluorescence intensity (CMFI) was calculated using the formula [Integrated Density − (Area of selected region × Mean fluorescence of background)]. Shapiro–Wilk test was used to test for normal distribution, and if data is normally distributed, a two-tailed unpaired Student's *t* test with false discovery rate (FDR) adjustment for multiple comparisons (two-stage step-up; Benjamini, Krieger, and Yekutieli) was used to compare means. If data was not normally distributed, a Mann–Whitney *U* test with FDR adjustment for multiple comparisons (two-stage step-up; Benjamini, Krieger, and Yekutieli) was used to compare the mean ranks for *Prdm16*-cKO cell densities, cochlear sensory epithelium length, MCFI, structural morphometric measurements, and volumes relative to littermate heterozygote controls. 95% confidence intervals were calculated: $\mathrm{CI}=\bar{x}\pm z\frac{s}{\sqrt{n}}$. For ABR data analysis, Shapiro–Wilk test was used to test for normal distribution, and Kruskal–Wallis test followed by Dunn's test for multiple comparisons was used to compare mean ranks. The number of animals (*n*), male and female littermates, were used. The *p* values are reported for each experiment.

## Results

### Generation and validation of *Prdm16* cKO mouse model

**Figure 1.**
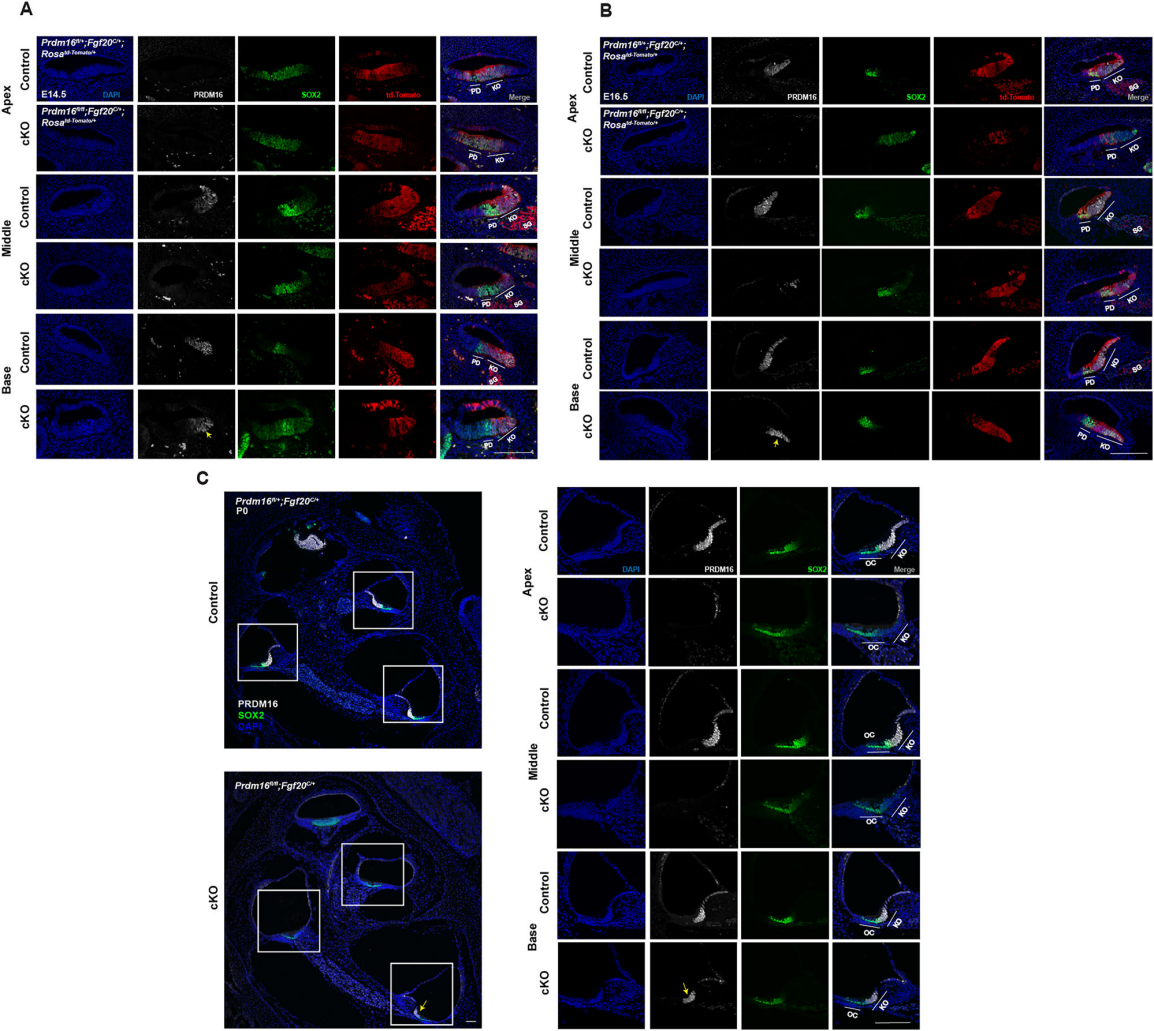
Generation and validation of *Prdm16* cKO mice. ***A***, Immunostaining of cochlear sections from *Fgf20^cre/+^*; *Prdm16^fl/fl^*; *Rosa^tdTomato/+^* (cKO) and littermate controls from E14.5 embryos (***A***) and E16.5 embryos (***B***) showing *Fgf20Cre*-mediated recombination and PRDM16 expression in control versus cKO cochlea. Yellow arrows indicate persistent PRDM16 expression in the basal turn of *Prmd16* cKO (PRDM16 in white, SOX2 in green, and td-Tomato in red, *n* = 3–5). ***C***, Immunostaining of cochlear sections from P0 *Prdm16* cKO and littermate heterozygote controls showing PRDM16 expression (white) and SOX2 (green) in different cochlear turns (*n* = 3). Magnified areas are marked with white boxes. Yellow arrow indicates persistent PRDM16 expression in the basal turn of *Prmd16* cKO. PD, prosensory domain; SG, spiral ganglion; OC, organ of Corti; KO, Kölliker's organ (scale bar, 100 µm).

First, we generated a *Prdm16* conditional deletion mouse model utilizing *Fgf20*-Cre line (Huh et al., 2015) and *Prdm16^fl^* line (Cohen et al., 2014). To validate the timing, domain, and efficiency of *Fgf20* Cre-mediated *Prdm16* deletion, we utilized the reporter strain *Rosa^td-Tomato^*. We generated *Fgf20^cre/+^*; *Prdm16^fl/fl^*; *Rosa^tdTomato/+^* embryos and analyzed cochlear sections at E14.5 and E16.5 time points. *Fgf20-*Cre activity (as indicated by td-Tomato red fluorescence) appeared within the prosensory domain, Kölliker's organ, and spiral ganglion at both time points (Fig. 1*A*,*B*; *n* = 3–5). To validate *Prdm16* deletion efficiency in conditional mutant cochleae, we immunostained cochlear sections at E14.5, E16.5, and P0 with anti-PRDM16 antibody that showed loss of staining in the middle and apical turns compared with littermate controls (Fig. 1*A–C*; *n* = 3–5). The Kölliker's organ within the basal turn showed some PRDM16 expression in *Prdm16* cKO cochlea, indicating that this region has partially escaped the CRE-mediated recombination. Nevertheless, *Prdm16* was efficiently deleted from the rest of the cochlear duct's Kölliker's organ.

### *Prdm16* cKO cochlear phenotype at birth

**Figure 2.**
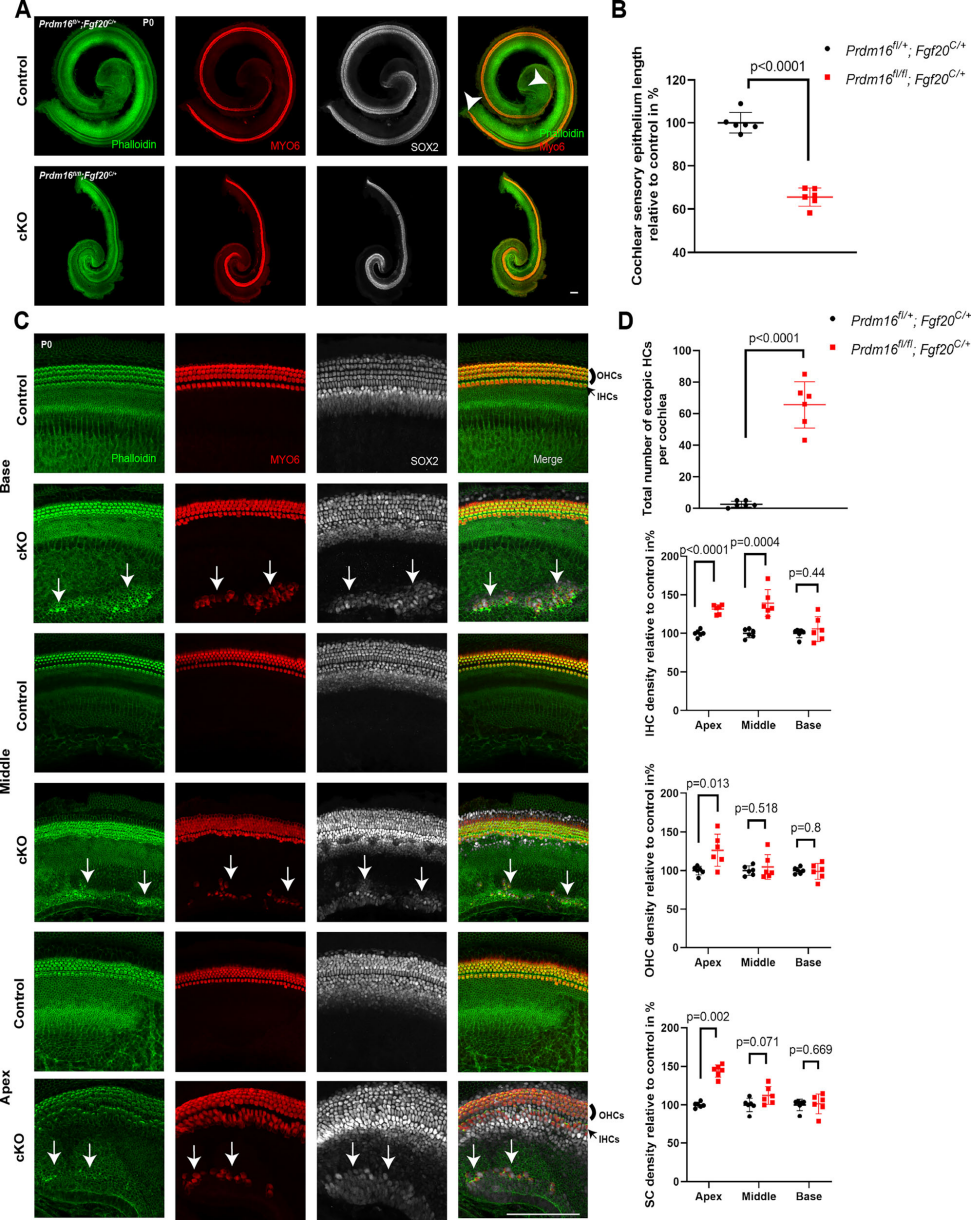
*Prdm16* regulates cochlear lengthening and restricts sensory fates in developing Kölliker's organ. ***A***, Immunostained whole mount cochlear epithelium from P0 *Prdm16* cKO and littermate heterozygote controls showing shortening of the cochlear duct. Arrowheads refer to the beginning and end of the cochlear duct length measurement. ***B***, Quantification of cochlear duct length (*n* = 6/group, mean ± SD, multiple unpaired two-tailed Student's *t* test, *p* value as indicated). ***C***, Immunostained whole mount cochlear epithelium stained with phalloidin for F-actin enrichment in hair bundles, MYO6 for hair cells, and SOX2 for supporting cells showing the presence of ectopic islands of sensory epithelia within the Kölliker's organ (white arrows), increased density of epithelial cells in *Prdm16* cKO apical turn compared with littermate heterozygote controls including inner hair cells, outer hair cells, and supporting cells. ***D***, Quantification and statistical analysis (*n* = 6/group, mean ± SD, multiple unpaired two-tailed Student's *t* test or Mann–Whitney *U* test, *p* value as indicated). OHCs, outer hair cells; IHCs, inner hair cells; SC, supporting cells. Scale bar = 100 µm.

We analyzed the cochlear phenotype of *Prdm16* cKO at P0 through whole-mount cochlear epithelium immunostaining using MYO6 hair cell marker and SOX2 supporting cell marker. Data shows shortening of the cochlear sensory epithelium as indicated by the beginning and end of the sensory epithelium staining (66% compared with control; Fig. 2*A*,*B*; *n* = 6/group, unpaired two-tailed Student's *t* test, *p* value as indicated). We noticed the presence of ectopic islands of sensory epithelia within the Kölliker's organ that contained both MYO6+ and SOX2+ cells in *Prdm16* cKO cochleae (Fig. 2*C*). The total number of ectopic MYO6+ cells in *Prdm16* cKO cochleae ranged from 40 to 87 with a mean of 65 cells per cochlea and were present in scattered clusters within the Kölliker's organ (Table 1; Fig. 2*C*,*D*; *n* = 6/group, unpaired two-tailed Student's *t* test, *p* value < 0.05). We observed an increased density of epithelial cells in *Prdm16* cKO apical turn compared with littermate heterozygote controls, including inner hair cells (31% increase), outer hair cells (26% increase), and SOX2+ supporting cells (41% increase; Table 1; Fig. 2*C*,*D*; *n* = 6/group, multiple unpaired two-tailed Student's *t* test or Mann–Whitney *U* test, *p* value as indicated). No statistically significant changes were observed in the base or the middle turns (except for the IHC density that showed an increase in the middle turn).

**Table 1.** P0 cochlear epithelium cell densities in *Prdm16* cKO compared with control

	Control*	*Prdm16* cKO*	FC relative to control	*p* value
Cochlear sensory epithelium length in µm	4,632.3 ± 160.3	3,039 ± 142.8	0.66	<0.0001**
Total number of ectopic HCs per cochlea	2.6 ± 1.4	65.5 ± 10.7	25.19	<0.0001**
IHC density in the base/100 µm	12.6 ± 0.6	13.4 ± 1.6	1.06	0.44**
IHC density in the middle/100 µm	14.4 ± 0.6	20.1 ± 2	1.40	0.0004**
IHC density in the apex/100 µm	16.2 ± 0.5	21.2 ± 0.8	1.31	<0.0001**
OHC density in the base/100 µm	42.6 ± 1.4	42.1 ± 3.5	0.99	0.8**
OHC density in the middle/100 µm	46.1 ± 2.3	48.2 ± 5.9	1.05	0.518**
OHC density in the apex/100 µm	49.3 ± 2.2	62.3 ± 8.2	1.26	0.013**
SC density in the base/100 µm	86.8 ± 5.1	88 ± 8.9	1.01	0.669***
SC density in the middle/100 µm	89.7 ± 6.2	100.8 ± 8.3	1.12	0.071***
SC density in the apex/100 µm	101.8 ± 2.8	144 ± 6.4	1.41	0.002***

FC, fold change.

* Means ± 95% confidence interval (CI), *N* = 6/group.

** Unpaired two-tailed Student's *t* test.

*** Mann–Whitney *U* test.

### *Prdm16* cKO cochlear phenotype during early postnatal development

**Figure 3.**
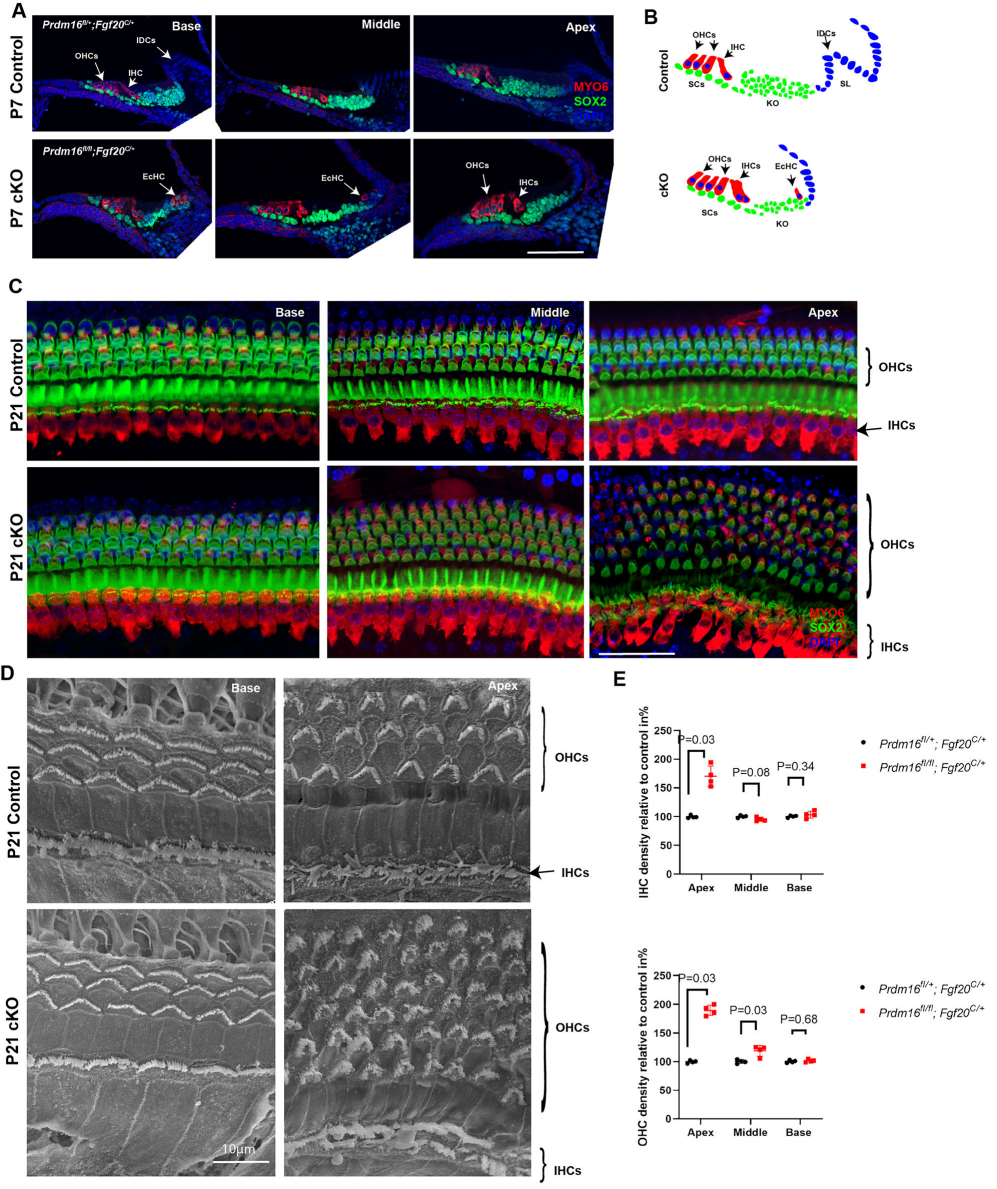
*Prdm16* cKO cochlear phenotype persists through postnatal maturation. ***A***, Immunostained cochlear sections from P7 *Prdm16* cKO and littermate heterozygote controls showing MYO6 + ectopic hair cells (EcHCs) in the region of interdental cells (IDCs) as well as multiple rows of outer hair cells in the middle and apical turns consistent with P0 phenotype (*n* = 4/group). ***B***, Schematic diagram recapitulating the phenotype described in ***A***. ***C***, Immunostained whole mount cochlear epithelium from P21 *Prdm16* cKO and littermate heterozygote controls showing increased OHC density in middle and apical turns, as well as increased IHC density in apical turn in *Prdm16* cKO cochlea. ***D***, Scanning electron microscopic images from P21 *Prdm16* cKO and littermate heterozygote control cochleae showing immature bundle morphology in the apical turn of *Prdm16* cKO (*n* = 3/group). ***E***, Quantification and statistical analysis of OHC and IHC densities (*n* = 4/group, mean ± SD, Mann–Whitney *U* test, *p* value as indicated). OHCs, outer hair cells; IHCs, inner hair cells; EcHCs, Ectopic hair cells; SCs, supporting cells; IDCs, interdental cells; KO, Kölliker's organ; SL, spiral limbus. Scale bar: 100 µm unless otherwise specified.

Since *Fgf20Cre*-driven *Prdm16* deletion did not affect pups’ survival, we utilized this model to investigate postnatal cochlear development in *Prdm16-*defecient mice. We analyzed P7 cochleae by immunostaining for MYO6 and SOX2 markers. Results from *Prdm16* cKO showed extra rows of inner and outer hair cells in both the middle and apical turns as well as ectopic MYO6+ cells within the region of the interdental cells (IDCs; Fig. 3*A*,*B*; *n* = 3/group). Analysis of P21 cochlear epithelium revealed increased hair cell densities in *Prdm16* cKO apical turn compared with littermate heterozygote controls, including inner hair cells (69% increase) and outer hair cells (91% increase;*n* = 4/group, Mann–Whitney *U* test, *p* value as indicated; Table 2, Fig. 3*C–E*). Interestingly, no ectopic MYO6+ cells were observed at P21, indicating failure of these cells to survive. Scanning electron microscopy (SEM) showed similar results to the immunostaining analysis. Additionally, SEM showed evidence of immature stereocilia bundle morphology in the apical turn of *Prdm16* cKO cochlea (Fig. 3*D*; *n* = 3/group).

**Table 2.** P21 cochlear hair cell densities in *Prdm16* cKO compared with control

	Control*	*Prdm16* cKO*	FC relative to control	*p* value**
IHC density/100 µm				
Apex	12.77 ± 0.26	21.62 ± 3.17	1.69	0.03
Middle	14.13 ± 0.39	13.44 ± 0.66	0.95	0.08
Base	13.64 ± 0.46	14.14 ± 1.76	1.04	0.34
OHC density/100 µm				
Apex	41.36 ± 1.41	78.95 ± 4.88	1.91	0.03
Middle	40.86 ± 1.87	50.5 ± 1.1	1.24	0.03
Base	39.49 ± 1.2	40.5 ± 1	1.03	0.68

FC, fold change; IHC, inner hair cell; OHC, outer hair cell.

* Means ± 95% CI.

** *n* = 4/group, Mann–Whitney *U* test.

### *Prdm16* overexpression is not sufficient to inhibit hair cell fates in ex vivo cultures

**Figure 4.**
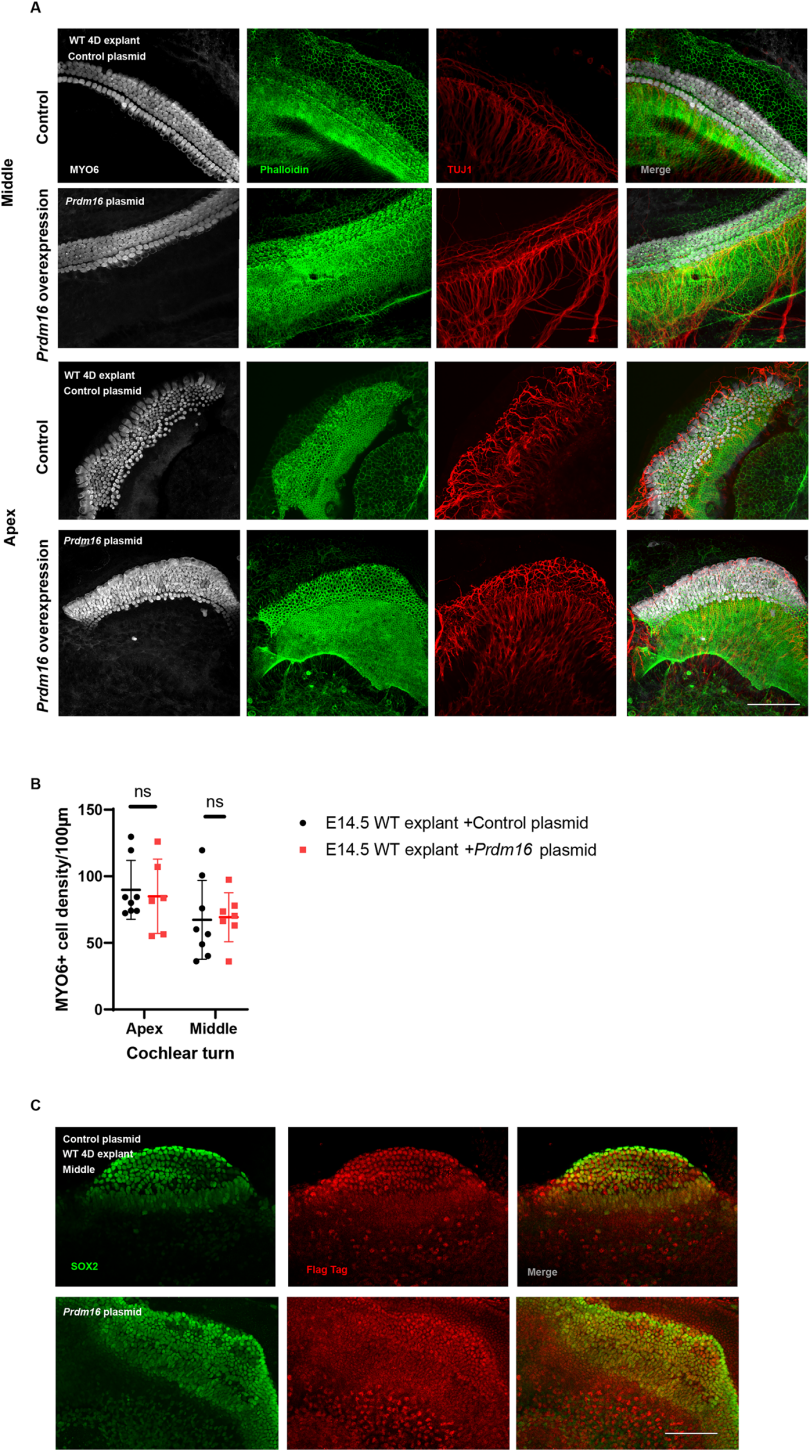
*Prdm16* overexpression is not sufficient to inhibit sensory fates in vitro. ***A***, E14.5 WT cochlear explants 4 d post electroporation with *Prdm16*-expressing or control plasmids. Cultures are immunostained with phalloidin for F-actin enrichment in hair cell bundles (green), MYO6 for hair cells (white), and TUJ1 for neurons (red). No significant changes are noticed in the sensory epithelium. ***B***, Quantification of MYO6+ cells in apical and middle turns of the explants (*n* = 6–8/group, mean ± SD, multiple unpaired two-tailed Student's *t* test, *p* value as indicated). ***C***, Immunostained explants stained with FLAG antibody for plasmid expression, and SOX2 for supporting cells showing colocalization. Scale bar, 100 µm.

Since we identified ectopic sensory epithelium within the Kölliker's organ of *Prdm16* cKO cochleae, we sought to investigate whether *Prdm16* overexpression can inhibit sensory fates within the prosensory domain during cochlear development. We collected WT E14.5 cochlear tissue including the epithelium, surrounding mesenchyme and spiral ganglia, then electroporated the tissues with either control plasmid or *Prdm16* expression plasmid. After 4 d in vitro, immunostaining for MYO6 (hair cell marker), phalloidin (F-actin enriched hair bundles staining), and TUJ1 (spiral ganglion neuronal marker) revealed no significant changes in hair cell density or hair cell innervation patterns (Fig. 4*A*,*B*; *n* = 6–8/group, multiple unpaired two-tailed Student's *t* test, *p* value as indicated). To confirm plasmid expression in the cochlear cultures, we performed immunostaining for FLAG tag (expressed by both plasmids) that showed expression within the epithelium in both control and *Prdm16* overexpression cultures (Fig. 4*C*).

### *Prdm16* is required for spiral limbus and tectorial membrane structure

**Figure 5.**
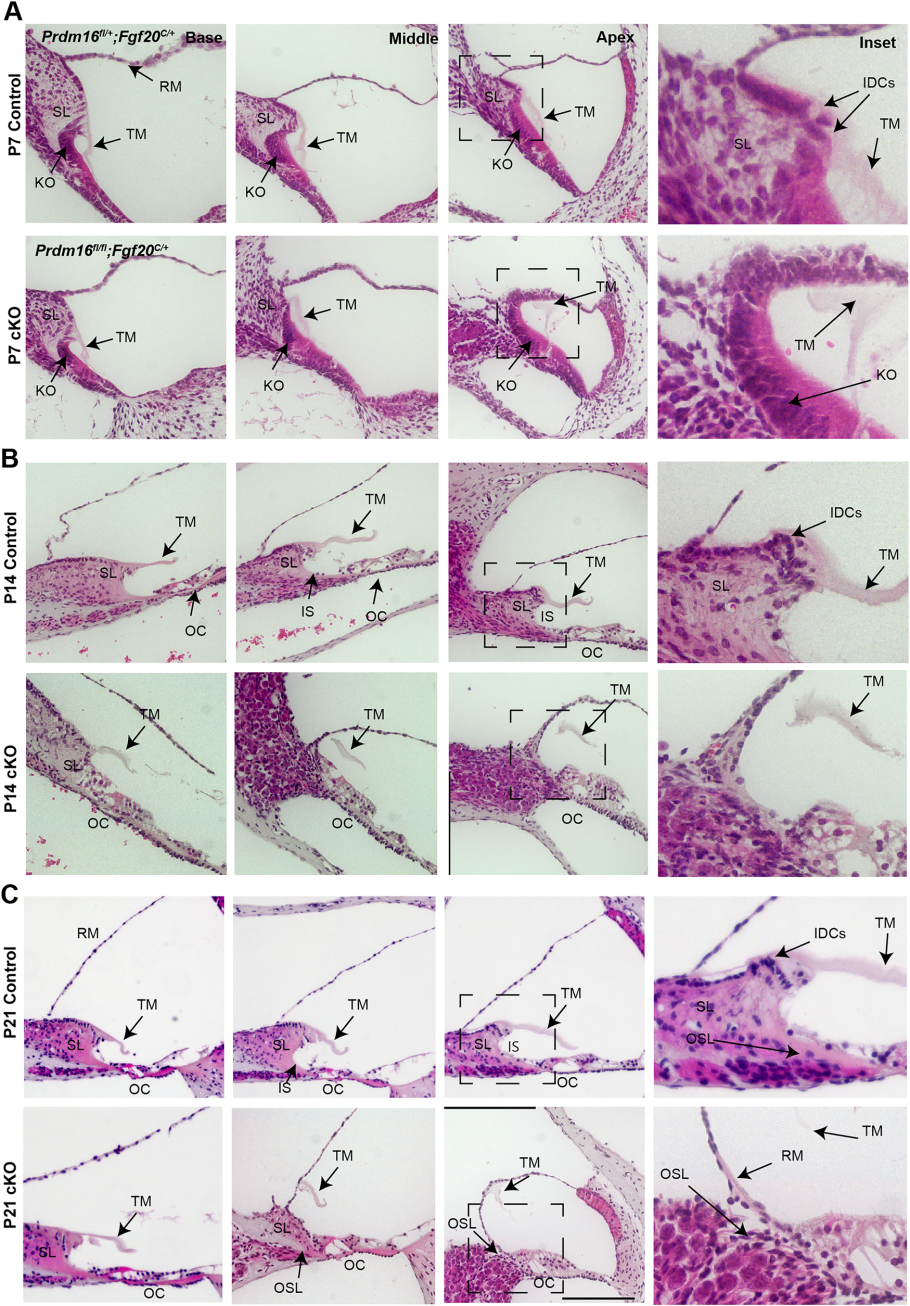
*Prdm16* is required for spiral limbus and tectorial membrane structure as evident by hematoxylin and eosin (H&E) staining. H&E-stained cochlear sections from P7 (***A***), P14 (***B***), and P21 (***C***) *Prdm16* cKO and littermate heterozygote controls (*n* = 3/group). Images show hypoplastic spiral limbus (SL), lack of inner sulcus (IS), lack of interdental cells (IDCs), and abnormal attachment of tectorial membrane (TM) to the Reissner's membrane (RM) in the middle and apical turns in *Prdm16* cKO cochleae. Dashed boxes indicate magnified insets. OC, Organ of Corti; KO, Kölliker's organ; PD, prosensory domain; SV, stria vascularis; TM, tectorial membrane; SL, spiral limbus; RM, Reissner's membrane; OSL, osseous spiral lamina; IS, inner sulcus; IDCS, interdental cells. Scale bar, 200 µm.

To investigate the structural defects in *Prdm16* cKO cochlea, we utilized H&E staining of cochlear sections from P7, −14, and −21 (*n* = 3/group/time point). In *Prdm16* cKO cochlea, the spiral limbus failed to develop in the middle and apical turns (Fig. 5*A–C*). Furthermore, interdental cells were not evident in the middle and apical turns. The lack of a spiral limbus and interdental cells led to the direct continuation of Reissner's membrane and the organ of Corti epithelium at P21 (Fig. 5*C*). The tectorial membrane in the middle and apical turns lost its modiolar attachment (possibly due to a lack of interdental cells and spiral limbus). Instead, it adhered to the Reissner's membrane at P7–P21 (Fig. 5*A–C*).

**Figure 6.**
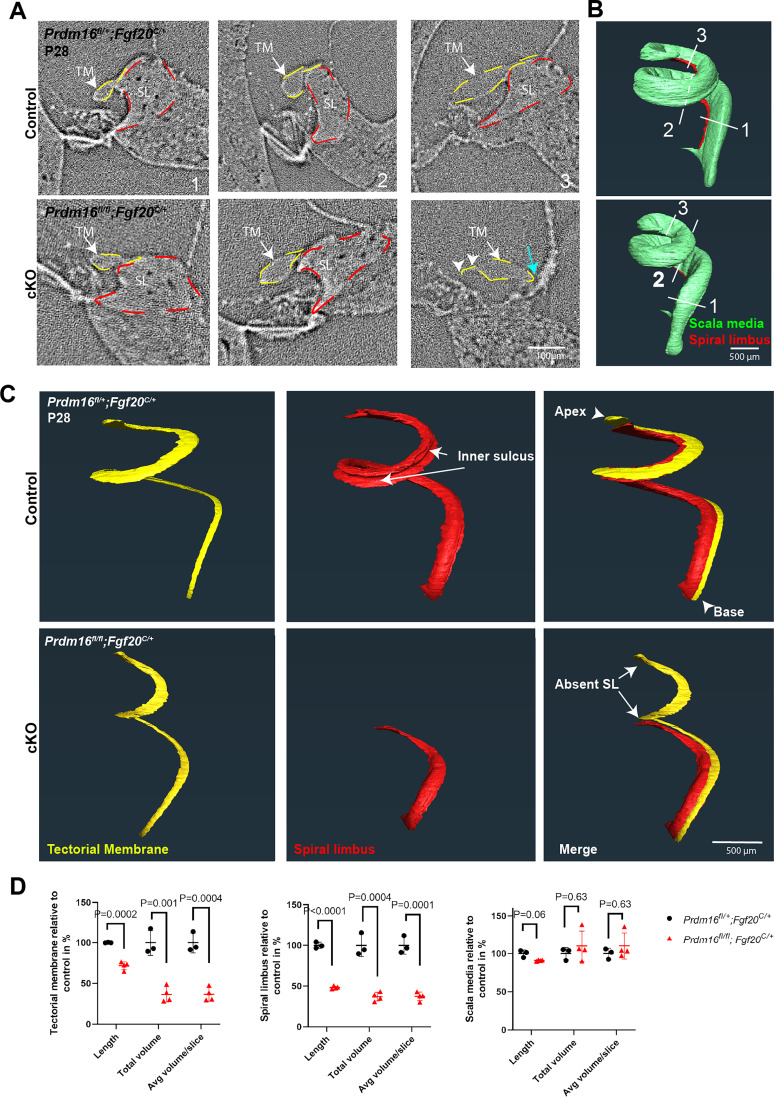
*Prdm16* is required for normal spiral limbus and tectorial membrane structure, as synchrotron x-ray μCT shows. ***A***, Representative μCT images showing tectorial membrane (TM) outlined in dashed yellow line and spiral limbus (SL) outlined in dashed red line in the base, middle, and apical turns (levels indicated as 1, 2, and 3, respectively on the 3D reconstructed surface model). ***B***, 3D reconstruction of scala media (in green) and spiral limbus (in red) showing levels indicated in (***A***). In the apical turn, *Prdm16* cKO cochlea shows loss of spiral limbus, abnormal attachment of the tectorial membrane to Reissner's membrane (blue arrow) and thinning of the marginal domain of the tectorial membrane (white arrowheads). ***C***, 3D surface reconstruction using Amira of the cochlear spiral limbus (SL in red) and tectorial membrane (TM in yellow) demonstrating structural changes in the middle and apical regions of *Prdm16* cKO cochlea including loss of spiral limbus, lack of inner sulcus, and shortening of tectorial membrane. ***D***, Quantification of the tectorial membrane, spiral limbus, and scala media volume and length (*n* = 3–4/group, mean ± SD, multiple unpaired two-tailed Student's *t* test or Mann–Whitney *U* test, *p* value indicated). Scale bar as indicated.

To circumvent tectorial membrane susceptibility to histological processing artifacts (specifically dehydration) and to reliably measure tectorial membrane and spiral limbus volume changes in *Prdm16* cKO cochlea, we utilized synchrotron x-ray microcomputed tomography (μCT) to analyze nondehydrated P28 inner ears from *Prdm16* cKO and littermate heterozygote controls. Segmentation of different cochlear structures (including spiral limbus, tectorial membrane, and scala media) in μCT slices based on differential densities (Fig. 6*A*) and subsequent surface generation using Amira software (Fig. 6*B*,*C*) enabled a thorough analysis of different structures’ morphology and volume. Average lengths and volumes of analyzed structures can be found in Table 3 (*n* = 3–4/group, multiple unpaired two-tailed Student's *t* test or Mann–Whitney *U* test, *p* value as indicated). Analysis of *Prdm16* cKO tectorial membrane revealed shortening (72% compared with control), reduced total volume (36% compared with control), and reduced average volume per slice (37% compared with control), indicating a significant loss in the tectorial membrane matrix volume (Table 3; Fig. 6*A–D*; *n* = 3–4/group, unpaired two-tailed Student's *t* test, *p* value as indicated). An abnormal anchorage of the tectorial membrane to Reissner's membrane in the apical half of the cochlea was noticed in *Prdm16* cKO cochlea (Fig. 6*A*). The marginal domain of tectorial membrane (Goodyear and Richardson, 2018) in the apical turn of *Prdm16* cKO cochlea showed significant thinning (Fig. 6*A*). Quantification of spiral limbus attributes showed a reduction in total length (48% compared with control), total volume (37% relative to controls), and average volume per slice (37% compared with control; Table 3; Fig. 6*A–D*; *n* = 3–4/group, unpaired two-tailed Student's *t* test, *p* value as indicated). The total length of the scala media was 91.5% compared with the control, with no significant reduction in total volume or volume per slice (Table 3, Fig. 6*D*). Then, 360° rotation movies for surfaces of representative samples from both control and *Prdm16* cKO cochlea can be found in Movies 1–8.

**Table 3.** P28 cochlear morphometric measurements in *Prdm16* cKO compared with control

	Control*	*Prdm16* cKO*	FC relative to control	*p* value
Tectorial membrane				
Average length in µm	5,431 ± 22	3,895 ± 265	0.72	0.0002**
Average total volume in µl	2.10 × 10^−2^ ± 3.73 × 10^−3^	7.67 × 10^−3^ ± 1.93 × 10^−3^	0.36	0.0017**
Average volume/slice in µl	1.62 × 10^−5^ ± 2.29 × 10^−6^	5.91 × 10^−6^ ± 1.32 × 10^−6^	0.37	0.0004**
Spiral limbus				
Average length in µm	4,436 ± 190	2,140 ± 76	0.48	<0.0001**
Average total volume in µl	3.97 × 10^−2^ ± 6.24 × 10^−3^	1.47 × 10^−2^ ± 2.08 × 10^−3^	0.37	0.0004**
Average volume/slice in µl	3.05 × 10^−5^ ± 3.76 × 10^−6^	1.14 × 10^−5^ ± 1.57 × 10^−6^	0.37	0.0001**
Scala media				
Average length in µm	8,019 ± 429	7,290 ± 60	0.91	0.06***
Average total volume in µl	4.04 × 10^−1^ ± 3.60 × 10^−2^	4.45 × 10^−1^± 8.07 × 10^−2^	1.10	0.63***
Average volume/slice in µl	3.12 × 10^−4^ ± 2.43 × 10^−5^	3.43 × 10^−4^ ± 5.35 × 10^−5^	1.10	0.63***

FC, fold change.

* Means ± 95% CI, *N* = 3–4/group.

** Unpaired two-tailed Student's *t* test.

*** Mann–Whitney *U* test.

### *Prdm16* is required for tectorial membrane collagen content and anchorage

**Figure 7.**
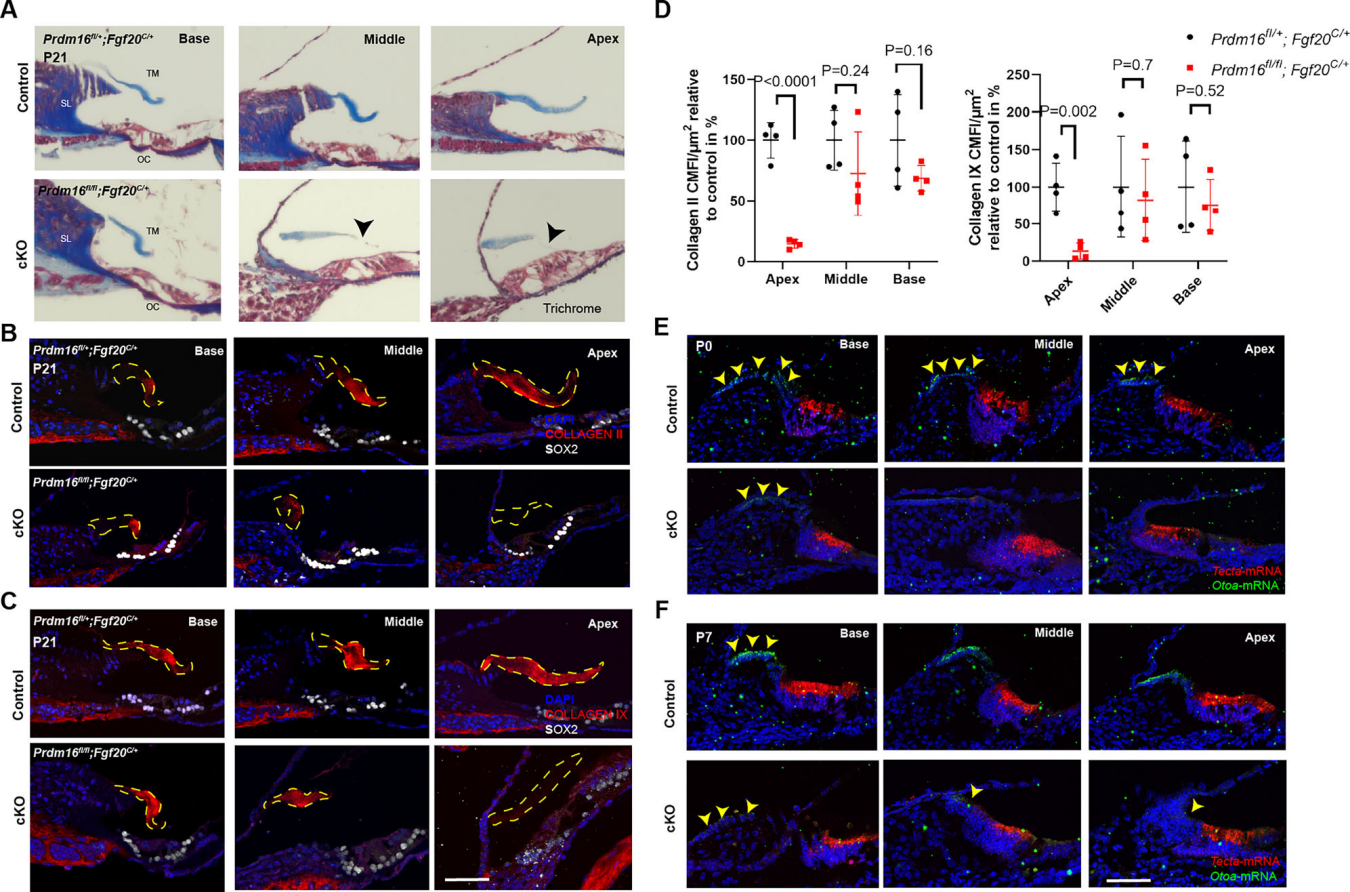
*Prdm16* is required for tectorial membrane anchorage and collagen. ***A***, Trichrome staining of cochlear sections from P21 *Prdm16* cKO and littermate heterozygote controls showing collagen fibers (blue), nuclei (black), and cytoplasm (red) demonstrating faint collagen staining in the tectorial membrane in *Prdm16* cKO cochlea as well as thinning of the marginal zone (black arrowheads). ***B***, ***C***, Immunostained cochlear sections from P21 *Prdm16* cKO and littermate heterozygote controls showing deficient COLLAGEN II and COLLAGEN IX immunostaining in the tectorial membrane in the apical turn of *Prdm16* cKO (tectorial membrane outlined with dashed lines). ***D***, Quantification of mean corrected fluorescence intensity (MCFI) of Collagen II and Collagen IX within the tectorial membrane in *Prdm16* cKO versus controls (*n* = 4/group, mean ± SD, multiple unpaired two-tailed Student's *t* tests, *p* value as indicated). ***E***, ***F***, Fluorescence in situ hybridization probing for *Otoa* and *Tecta* mRNAs in P0 and P7 cochlear sections from *Prdm16* cKO and littermate heterozygote controls showing loss of *Otoa* signal (a marker for interdental cells) from the middle and apical turns of *Prdm16* cKO cochlea (yellow arrowheads). SL, spiral limbus; TM, tectorial membrane; and OC, organ of Corti. Scale bar, 100 µm.

Since we observed a significant reduction in tectorial membrane volume (36% compared with control; Table 3) from μCT reconstruction in *Prdm16* cKO, we further analyzed tectorial membrane phenotype using collagen-specific staining techniques. Qualitative assessment of general collagen content within the tectorial membrane using trichrome staining at P21 showed faint collagen staining in *Prdm16* cKO tectorial membrane, with the marginal domain of the tectorial membrane lacking any collagen staining (Fig. 7*A*; *n* = 3/group). Immunostaining of cochlear sections with antibodies against previously identified tectorial membrane collagen proteins—including Collagen II and Collagen IX (Legan et al., 1997)—revealed significant reduction in the mean corrected fluorescence intensity (MCFI) for Collagen II (14.6% compared with controls) and Collagen IX (13.5% compared with controls) in the apical turn of *Prdm16* cKO tectorial membrane at P21 (*n* = 4/group, multiple unpaired two-tailed Student's *t* tests, *p* value aw indicated; Fig. 7*B–D*). In the basal and middle turns, collagen-specific staining was comparable with controls.

Since anchorage of the tectorial membrane to the interdental cells is mediated by the GPI-anchored glycoprotein otoancorin (OTOA; Zwaenepoel et al., 2002; Lukashkin et al., 2012), we performed mRNA fluorescence in situ hybridization to investigate *Otoa* mRNA expression changes in *Prdm16* cKO at P0 and P7. Interestingly, there was a complete loss of *Otoa* mRNA signal from the region of interdental cells in *Prdm16* cKO compared with control in the middle and apical turns (Fig. 7*E*,*F*), indicating a lack of differentiation of interdental cells in *Prdm16* cKO.

**Figure 8.**
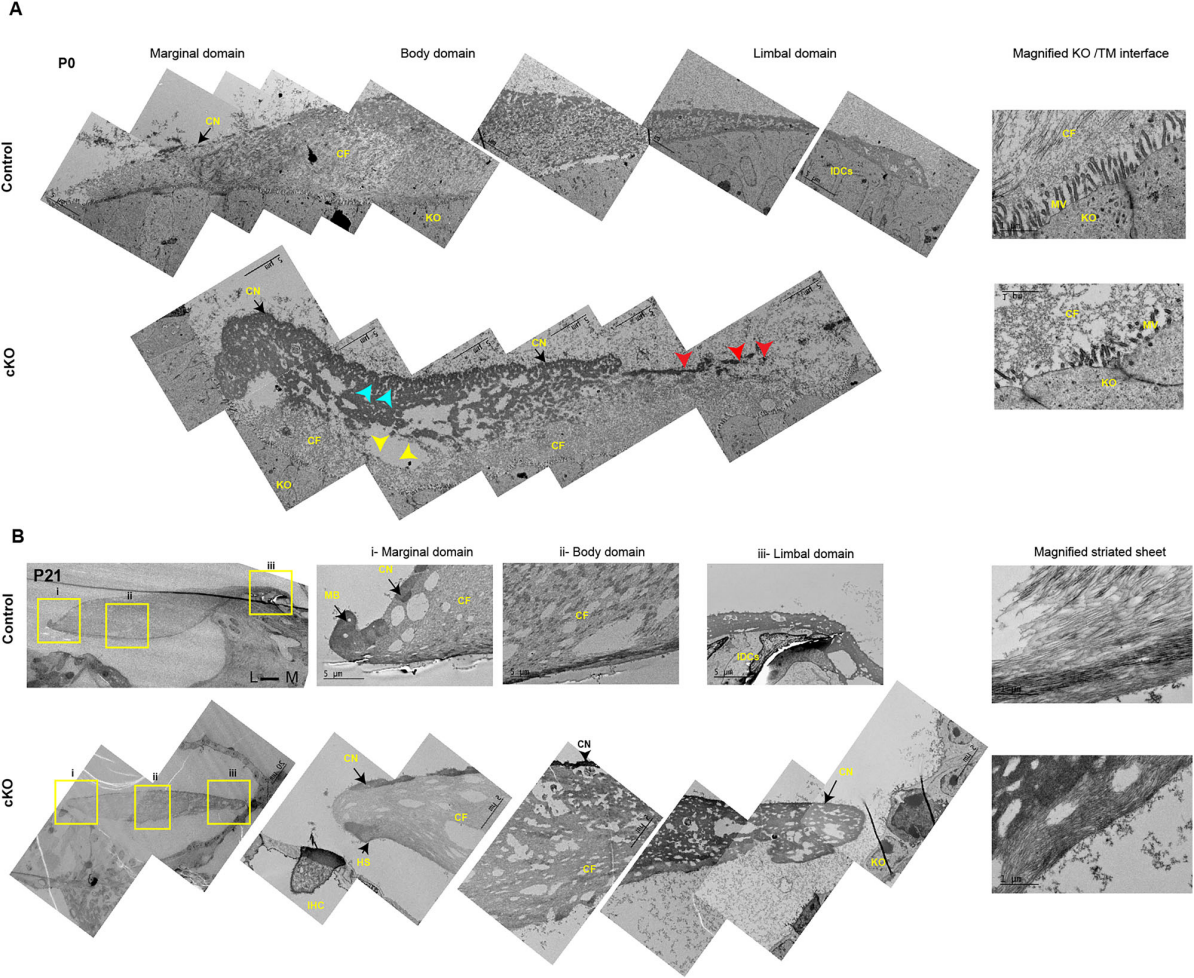
*Prdm16* regulates tectorial membrane maturation. ***A***, Transmission electron microscopic (TEM) images of P0 tectorial membrane showing different domains and the Kölliker's organ/tectorial membrane interface (KO/TM interface). P0 *Prdm16* cKO tectorial membrane shows absent limbal domain (red arrowheads), loose attachment of TM to the Kölliker's organ cells (yellow arrowheads), scarce collagen fibrils (CF), excessive glycoprotein aggregates (blue arrowheads) underneath the covernet (CN), and fewer microvilli (MV) in Kölliker's organ (KO) cells. ***B***, TEM images of the P21 tectorial membrane show different domains (marginal, body and limbal domains) and a magnified image for the striated sheet. *Prdm16* cKO tectorial membrane shows a lack of limbal domain, disrupted marginal band (MB), intact covernet (CN), and a modest amount of radially arranged collagen fibrils (CF). Scale bars as indicated. IHC, inner hair cell; HS, Hensen stripe; IDCs, interdental cells; KO, Kölliker's organ; MB, marginal band; CN, covernet; CF, collagen fibrils.

To characterize the ultrastructure of tectorial membrane in *Prdm16* cKO cochleae, we performed transmission electron microscopy at P0 and P21 (Fig. 8*A*,*B*; *n* = 3/group). At P0, the tectorial membrane in *Prdm16* cKO cochleae showed an absent limbal domain, loose separation from the Kölliker's organ cells, scarce collagen fibrils resembling mouse embryonic 14.5 matrix (Goodyear et al., 2017), and excessive glycoprotein aggregates underneath the covernet (Fig. 8*A*). Kölliker's organ cells showed fewer microvilli that were disconnected from the tectorial membrane matrix (Fig. 8*A*, magnified interface). At P21, *Prdm16* cKO tectorial membrane was detached, lacking the limbal domain, and showed a disrupted marginal band and a Hensen stripe-like structure overlying the inner hair cells (Fig. 8*B*). *Prdm16* cKO tectorial membranes showed a modest amount of collagen fibers that are radially arranged along the medial-lateral axis and an overlying covernet that is comparable with controls (Fig. 8*B*).

### *Prdm16* regulates spiral limbus development

**Figure 9.**
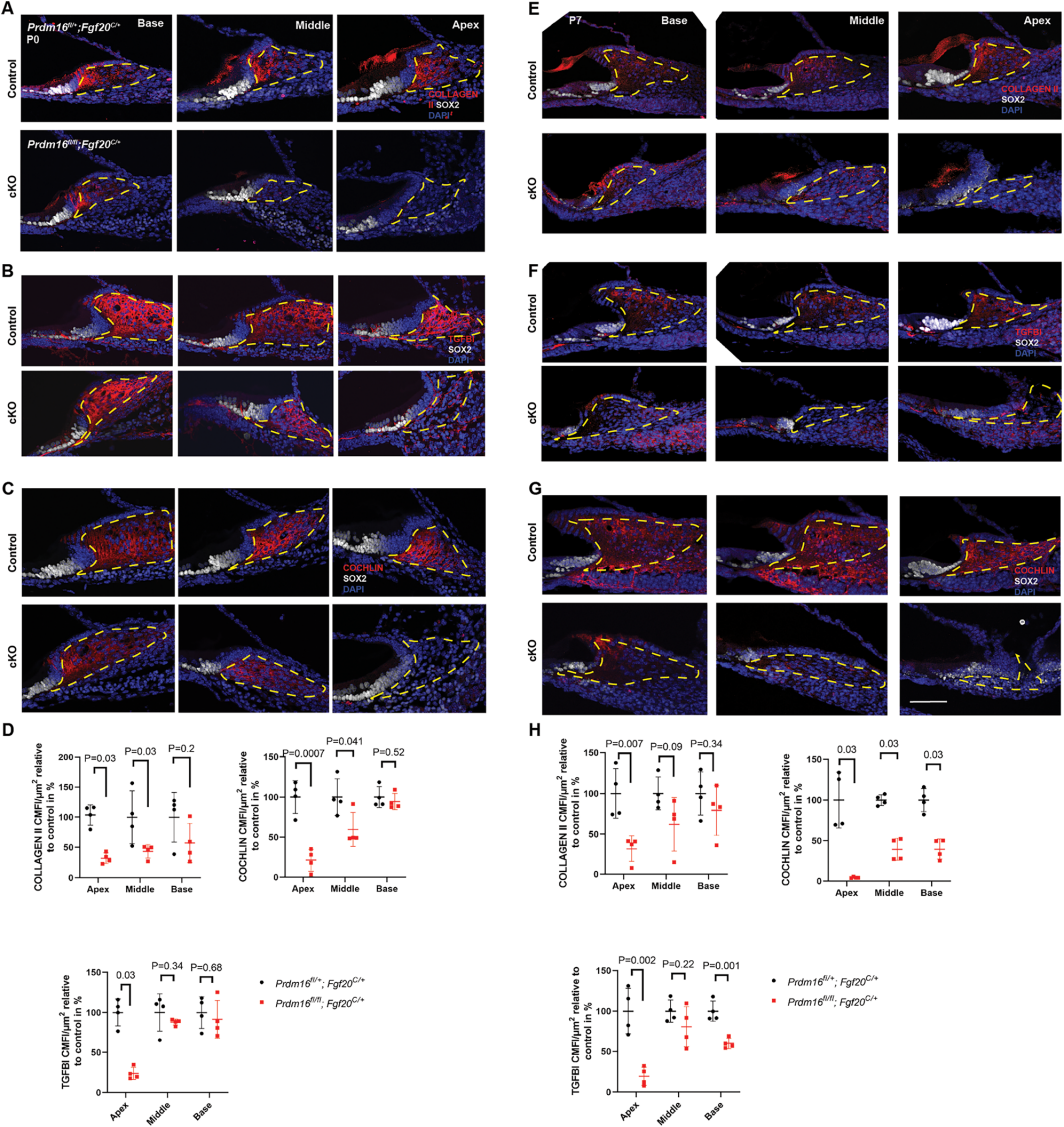
*Prdm16* expressed in Kölliker's organ regulates interstitial matrix within underlying spiral limbus mesenchyme. ***A–C***, Immunostaining of cochlear sections from *Prdm16* cKO mice at P0 for interstitial matrix proteins, including COLLAGEN II, TGFBI, and COCHLIN, showing reduced staining in the interstitial matrix of the spiral limbus in the apical turn of *Prdm16* cKO. ***D***, Quantification of mean corrected fluorescence intensity (MCFI) of interstitial matrix proteins within the spiral limbus in *Prdm16* cKO versus littermate heterozygote controls at P0 (*n* = 4/group, mean ± SD, multiple unpaired two-tailed Student's *t* tests or Mann–Whitney *U* test, *p* value as indicated). ***E–G***, Immunostaining of cochlear sections from *Prdm16* cKO mice at P7 for interstitial matrix proteins showing findings consistent with these at P0. ***H***, Quantification of mean corrected fluorescence intensity (MCFI) of interstitial matrix proteins within the spiral limbus mesenchyme in *Prdm16* cKO versus littermate heterozygote controls at P7 (*n* = 4/group, mean ± SD, multiple unpaired two-tailed Student's *t* tests or Mann–Whitney *U* test, *p* value as indicated). The dashed area outlines the spiral limbus (SL). Scale bar, 100 µm.

**Figure 10.**
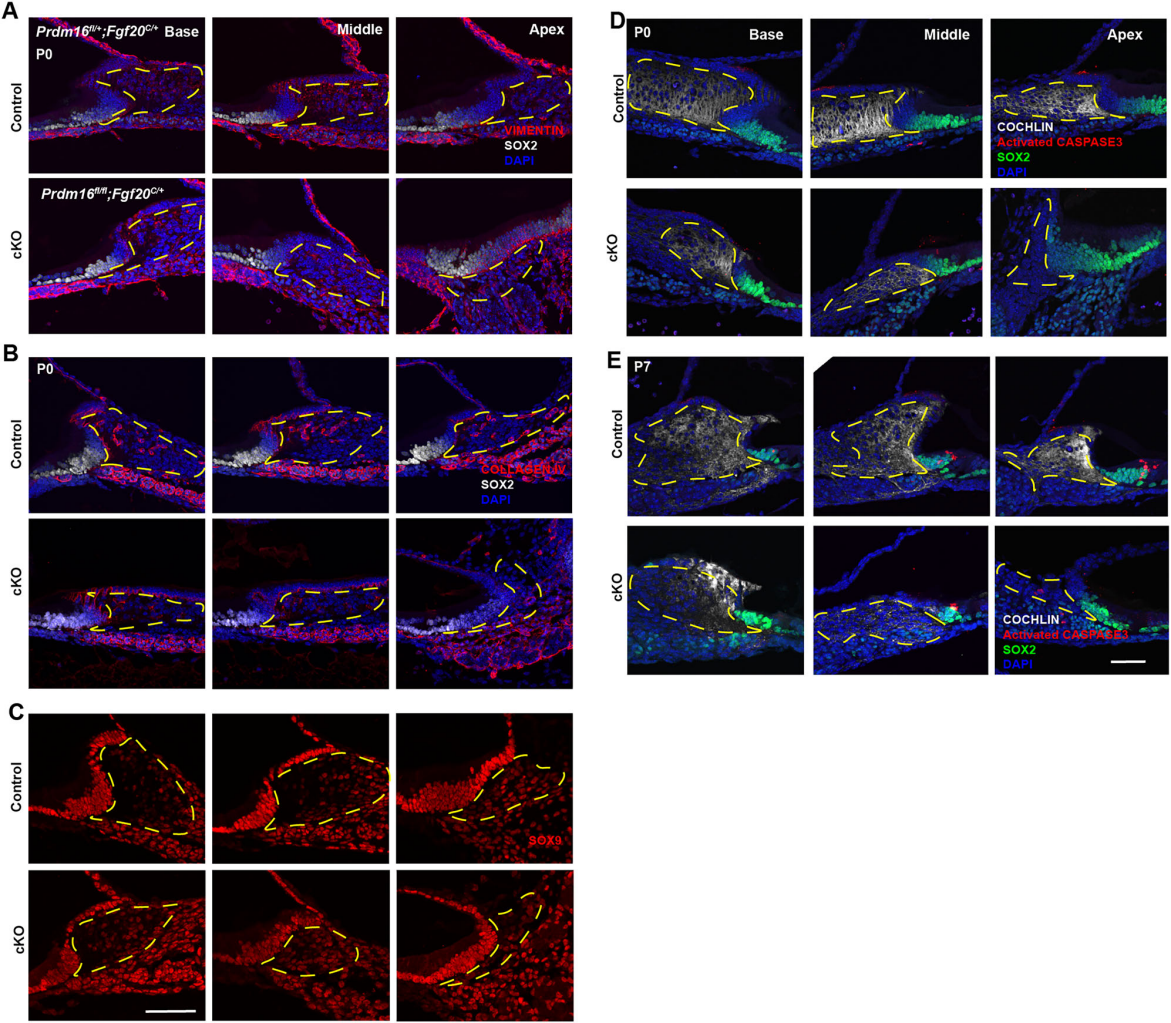
*Prdm16* deletion does not impact cell death in underlying spiral limbus mesenchyme. ***A***, ***B***, Immunostaining of cochlear sections from *Prdm16* cKO mice at P0 for extracellular matrix proteins, including VIMENTIN and COLLAGEN IV showing no difference in the extracellular matrix of the spiral limbus. ***C***, Immunostaining of cochlear sections from *Prdm16* cKO mice at P0 for SOX9 showing no considerable difference. ***D***, ***E***, Immunostaining of cochlear sections from *Prdm16* cKO mice at P0 and P7 for activated CASPASE 3 (apoptosis marker), along with COCHLIN (mesenchymal matrix marker), and SOX2 (supporting cell marker) showing no change in cell death within the spiral limbus mesenchyme. KO, Kölliker's organ. The dashed area outlines the SL; scale bar, 100 µm.

To understand the mechanism underlying the lack of the spiral limbus in the apical half of the cochlear duct in *Prdm16* cKO, we assessed spiral limbus mesenchymal interstitial matrix content. We examined multiple known interstitial matrix protein expression in the neonatal (P0) and early postnatal cochlea (P7) through immunostaining of cochlear sections using anti-COLLAGEN II, COLLAGEN IV, COCHLIN, VIMENTIN, and TGFBI antibodies (Slepecky et al., 1992; Thalmann, 1993; Tsuprun and Santi, 1999; Robertson et al., 2003; Rose et al., 2023). At P0, we observed reduced COLLAGEN II, COCHLIN, and TGFBI mean corrected fluorescence intensity in the extracellular matrix of the spiral limbus in the apical turn of *Prdm16* cKO cochleae (*n* = 4/group, multiple unpaired two-tailed Student's *t* tests or Mann–Whitney *U* test, *p* value as indicated; Fig. 9*A–D*). To investigate if these defects persist through postnatal development, we examined P7 and found consistent results (*n* = 4/group, multiple unpaired two-tailed Student's *t* tests for TGFBI and COLLAGEN II or Mann–Whitney *U* test for COCHLIN, *p* value as indicated; Fig. 9*E–H*). Other interstitial matrix proteins analyzed did not show change, including COLLAGEN IV (basement membrane enriched) and VIMENTIN (very weak expression in spiral limbus mesenchyme; Fig. 10*A*,*B*). Since SOX9 expression within the spiral limbus mesenchymal cells has previously shown to regulate spiral limbus interstitial matrix protein deposition (Trowe et al., 2010), we immunostained cochlear sections at P0 with anti-SOX9 antibody (Fig. 10*C*), yet we did not detect any change in the level of SOX9 in *Prdm16* cKO spiral limbus mesenchymal cells compared with controls, indicating that the defective matrix deposition in *Prdm16* cKO is not mediated through SOX9. We next analyzed cell death at P0 and P7 using activated CASPASE 3 antibody staining (McIlwain et al., 2013) and did not detect any change in cell death within the mesenchymal cells of *Prdm16* cKO compared with controls (*n* = 3/group; Fig. 10*D*,*E*), indicating noninvolvement of cell death in spiral limbus phenotype.

**Figure 11.**
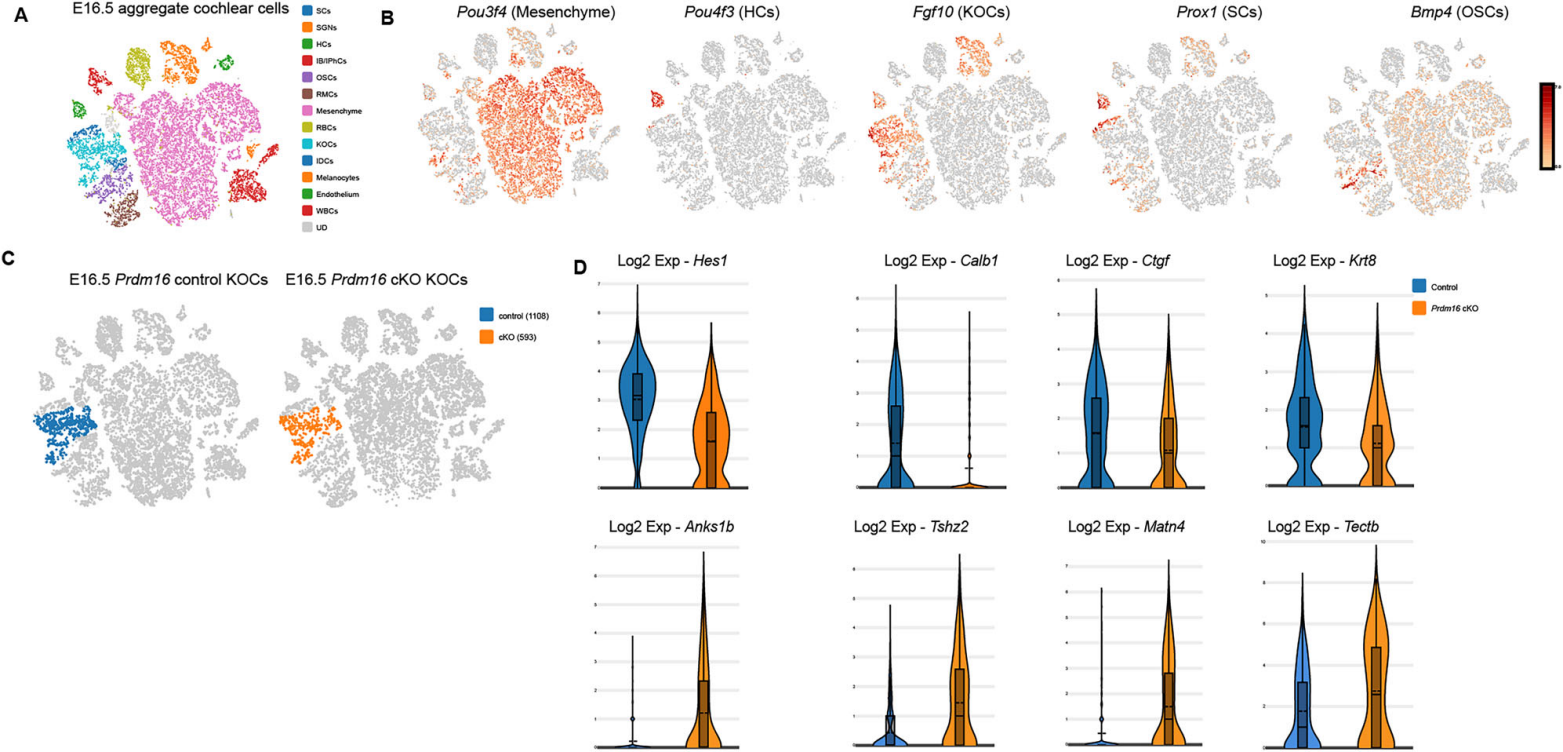
Single-cell RNA sequencing reveals differentially expressed genes in Kölliker's organ. ***A***, t-SNE plot representing graph-based clustering of single-cell gene expression aggregate data from both *Prdm16* cKO and control cochleae at E16.5 showing different cell clusters (color-coded). ***B***, Multiple t-SNE plots showing various marker gene expression per cluster (color represents enrichment level). ***C***, Split t-SNE plot representing clustered data from control versus *Prdm16* cKO cochlea at E16.5. Colored points indicate the transcriptome of Kölliker's organ cells with the total number of cells analyzed. ***D***, Violin plots showing a subset of differentially expressed genes within the Kölliker's organ (*n* = 3, pseudo-bulk method and sSeq, mean ± q3, *p* value < 0.05). SC, supporting cells; SGNs, spiral ganglion neurons; HCs, hair cells; IB/IphCs, inner border/inner phalangeal cells; OSCs, outer sulcus cells; RMCs, Reissner's membrane cells; RBCs, red blood cells; KOCs, Kölliker's organ cells; IDCs, interdental cells; WBCs, white blood cells; UD, unidentified cells.

Failure of spiral limbus development in *Prdm16* cKO suggests a non-cell autonomous role of Kölliker's organ epithelium in regulating underlying mesenchyme. To identify the epithelial-mesenchymal signaling required for spiral limbus development, we collected E16.5 control and *Prdm16* cKO cochleae, dissected the cochlear duct along with the surrounding mesenchyme, and preformed single-cell mRNA sequencing. We utilized the Loupe Browser (v.8) to cluster different cell populations from each sample based on the expression of known marker genes (Table 4; Fig. 11*A*,*B*). Kölliker's organ-specific differentially expressed genes in *Prdm16* cKO versus control were analyzed. Within the Kölliker's organ cells, we were able to identify multiple downregulated genes (Table 5; Fig. 11*C*,*D*). Out of the 12 downregulated genes (Log2 fold change; FC > 1, *p* value < 0.05), five genes were enriched in Kölliker's organ control cells (*Calb1*, *Ctgf*, *Hey1*, *Fam46a*, and *Galntl6*). Within the downregulated genes, we identified two genes that encode for secreted proteins (*Ctgf* and *Krt8*) with *Ctgf* to be the top downregulated gene coding for a secreted protein (Table 5; Fig. 11*C*,*D*).

**Table 4.** Marker genes used to identify cell clusters in single-cell mRNA sequencing

Marker gene symbol	Population	References
*Tbx18* and *Pou3f4*	Mesenchyme	Phippard et al., 1998; Trowe et al., 2008
*Fgf9*, *Otx1*, and *Otx2*	Roof epithelium	Morsli et al., 1999; Pirvola et al., 2004
*Prox1* and *Sox2*	Supporting cells	Bermingham-McDonogh et al., 2006; Hume et al., 2007
*Pou4f3*	Hair cells	Xiang et al., 1997
*Fgf10* and *Tecta*	Kölliker's organ	Pauley et al., 2003
*Bmp4* and *Lmx1a*	Outer sulcus cells	Morsli et al., 1998

**Table 5.** Differentially expressed genes in Kölliker's organ cells comparing *Prdm16* cKO to control samples at E16.5 (*n* = 3/group, Log2 FC > 1, *p* value < 0.05)

Downregulated gene symbol	Log2 fold change	*p* value*	Upregulated gene symbol	Log2 fold change	*p* value*
*Hes1*	−2.072874	5.59 × 10^−16^	*Anks1b*	2.729125	2.20 × 10^−14^
*Calb1*	−1.484853	1.14 × 10^−6^	*Tshz2*	1.804323	1.29 × 10^−9^
*Gm10076*	−1.308009	1.34 × 10^−6^	*Matn4*	1.667074	4.57 × 10^−5^
*Ctgf*	−1.282581	2.41 × 10^−5^	*Anxa5*	1.469127	2.17 × 10^−7^
*Uba52*	−1.266794	6.10 × 10^−6^	*Cpa6*	1.453544	4.76 × 10^−6^
*Krt8*	−1.230911	5.12 × 10^−5^	*Foxp2*	1.377273	7.03 × 10^−6^
*Fam46a*	−1.22214	3.73 × 10^−5^	*Ppp2r2b*	1.347479	1.67 × 10^−5^
*Hey1*	−1.196202	8.83 × 10^−5^	*Ephb1*	1.281419	3.73 × 10^−5^
*Galntl6*	−1.185235	0.000833	*Tectb*	1.194533	0.000351
*Id3*	−1.140664	7.12 × 10^−5^	*Tenm4*	1.072692	0.000245
*Gm42418*	−1.075368	0.000369	*Nrxn3*	1.00766	0.002703
*Cmss1*	−1.04359	0.000801			

* Exact negative binomial test (sSeq method).

**Figure 12.**
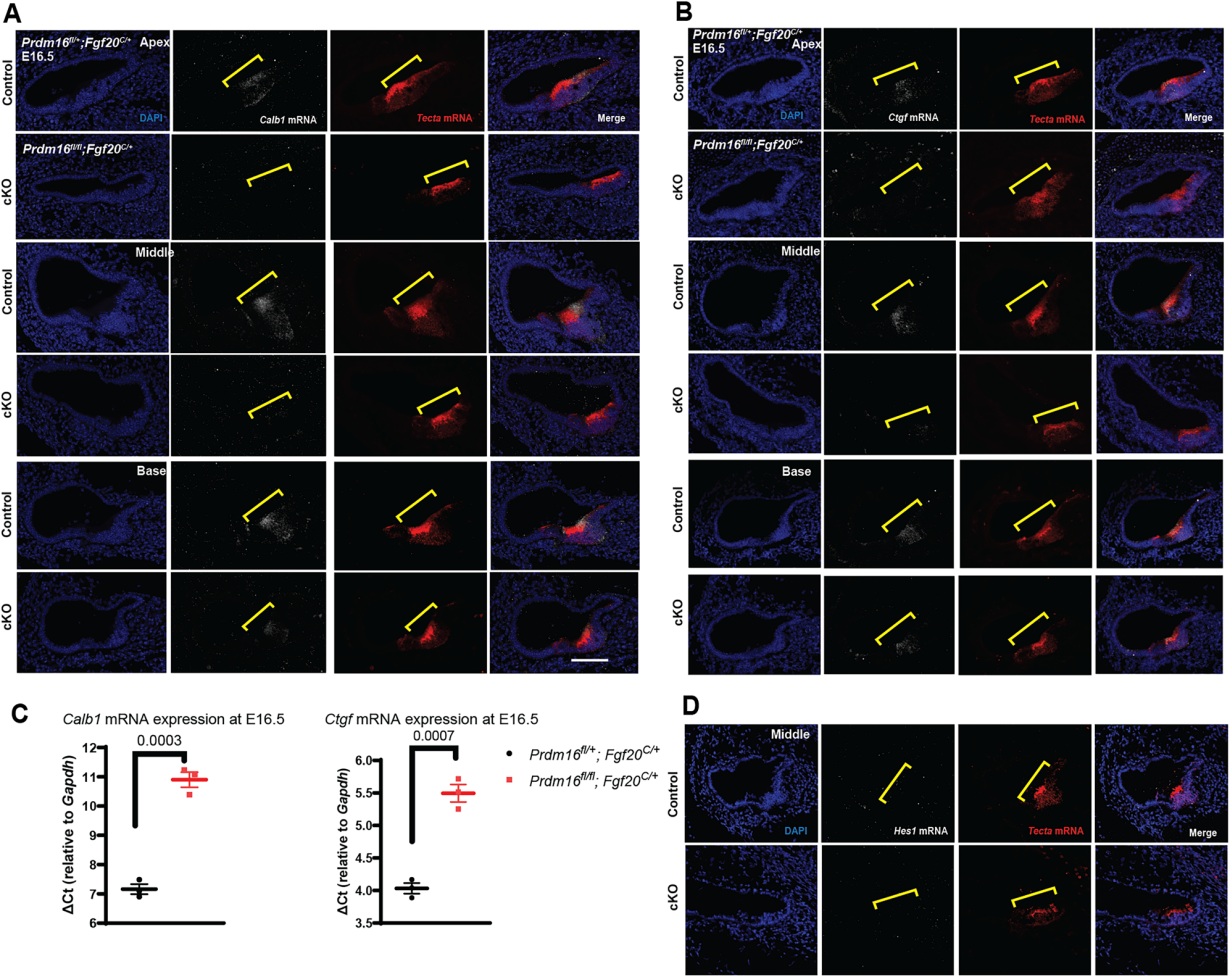
Validating differentially expressed genes within Kölliker's organ. Fluorescence in situ hybridization probing for *Calb1* mRNAs (***A***) and *Ctgf* mRNA (***B***) in E16.5 cochlear sections from *Prdm16* cKO and littermate heterozygote controls showing reduced *Calb1* and *Ctgf* signals within Kölliker's organ (yellow brackets) from the middle and apical turns of *Prdm16* cKO cochlea (*n* = 3–4/group). ***C***, Quantification of *Calb1* and *Ctgf* mRNA expression levels from microdissected cochlear ducts at E16.5 showing downregulation in *Prdm16* cKO compared with littermate heterozygote controls using qRT-PCR (*n* = 3/group, multiple unpaired two-tailed Student's *t* test, *p* value as indicated). ***D***, Fluorescence in situ hybridization probing for *Hes1* mRNAs in E16.5 cochlear sections from *Prdm16* cKO and littermate heterozygote controls showing no signal detection within Kölliker's organ in either group. Scale bar, 100 µm.

To validate differentially expressed genes in *Prdm16* cKO cochleae, we performed both fluorescence in situ hybridization (FISH) and quantitative real time PCR. Using FISH staining, we were able to confirm downregulation of *Calb1* and *Ctgf* as evident by reduced signal within the Kölliker's organ cells in *Prdm16* cKO cochleae including the middle and apical turns, but not in the basal turns at E16.5 (*n* = 3–4/group; Fig. 12*A*,*B*). Quantitative real-time PCR from microdissected cochlear epithelium showed 98% downregulation of *Calb1* mRNA and 65% downregulation of *Ctgf* mRNA in *Prdm16* cKO cochleae compared with controls at E16.5 (*n* = 3/group, multiple unpaired two-tailed Student's *t* test, *p* value as indicated; Fig. 12*C*). *Hes1* FISH was unable to detect *Hes1* mRNA transcripts in the epithelium of either control or *Prdm16* cKO cochleae (Fig. 12*D*).

### *Prdm16* is required for normal hearing in mice

**Figure 13.**
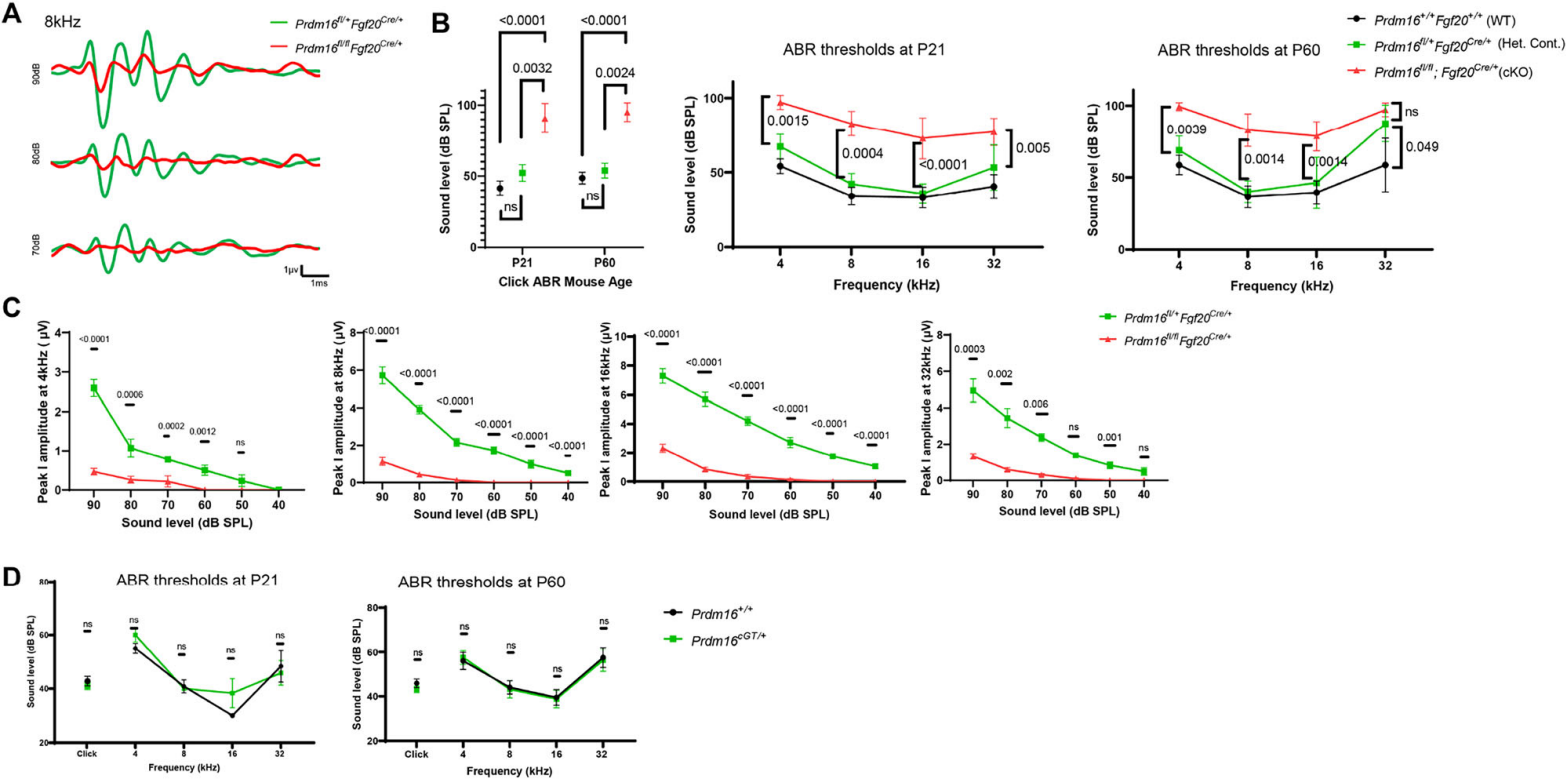
*Prdm16* is required for normal hearing in mice. ***A***, Auditory brainstem responses (ABRs) from P21 *Prdm16* cKO and littermate heterozygote controls using 8 kHz pure tone stimuli at 90, 80, and 70 dB SPL show reduced peak amplitudes. ***B***, ABR thresholds for click, 4, 8, 16, and 32 kHz pure tone stimuli at P21 and P60 showing statistically significant elevation in hearing thresholds in *Prdm16* cKO versus littermate heterozygote controls (*N* = 11–14/group at P21, *N* = 8–14 at P60; Kruskal–Wallis test followed by Dunn's test for multiple comparisons, mean ± SD, *p* value indicated). ***C***, First peak amplitude at 4, 8, 16, and 32 kHz analysis shows a statistically significant reduction in *Prdm16* cKO versus littermate heterozygote controls at P21 (*n* = 11–14/group, Mann–Whitney *U* test, mean ± SD, *p* value indicated). ***D***, ABR thresholds from *Prdm16^cGT/+^* and *Prdm16^+/+^* littermate controls at P21 and P60 showing no change in hearing thresholds (*N* = 6–8/group, Mann–Whitney *U* test, mean ± SD, *p* value < 0.5).

Given the significant structural defects in *Prdm16* cKO cochleae, we assessed hearing with ABRs in *Prdm16* cKO, littermate heterozygote controls, and wild-type age-matched animals with the same background at P21 and P60. Data shows statistically significant elevation in ABR thresholds in *Prdm16* cKO versus littermate heterozygote controls in click responses as well as pure tone stimuli across all tested frequencies at P21 (*n* = 11–14/group male and female, Kruskal–Wallis test followed by Dunn's test for multiple comparisons, adjusted *p* value as indicated), indicating a hearing deficit in *Prdm16* cKO (Fig. 13*A*,*B*). The biggest threshold shift was 40 dB at 8 kHz. *Prdm16* heterozygote control mice showed no significant ABR threshold differences when compared with WT age-matched littermates of a similar background at P21 (Fig. 13*B*). At P60, statistically significant elevations in ABR thresholds using click and tone stimuli were observed at 4, 8, and 16 kHz but not at 32 kHz in *Prdm16* cKO versus littermate heterozygote controls (Fig. 13*B*; *n* = 8–14/group, Kruskal–Wallis test followed by Dunn's test for multiple comparisons, adjusted *p* value as indicated). Comparing *Prdm16* heterozygote mice to WT age-matched littermates of a similar background at P60, no statistically significant difference is observed except at 32 kHz (Fig. 13*B*). Next, we analyzed ABR first peak amplitude at different frequencies and sound levels at P21. Data shows statistically significant decreases in first peak amplitude across all frequencies in *Prdm16* cKO animals versus heterozygote controls (*n* = 12–14/group, male and female, multiple Mann–Whitney *U* tests, adjusted *p* value as indicated; Fig. 13*C*). To confirm whether *Prdm16* haploinsufficiency is contributing to high frequency hearing loss in heterozygote mice, we utilized a different mouse model of *Prdm16* deletion (*Prdm16^cGT^*), which is maintained on FVB/NJ background. We analyzed ABR thresholds in *Prdm16^cGT/+^* versus *Prdm16^+/+^* littermate controls at P21 and P60. At both time points, no significant threshold shifts were observed (*n* = 7–8/group, male and female, Mann–Whitney *U* test, adjusted *p* value as indicated; Fig. 13*D*).

## Discussion

Previous studies have shown the transient nature of Kölliker's organ and its contribution to inner spiral sulcus and tectorial membrane development (Hinojosa, 1977; Anniko, 1980; Knipper et al., 1999; Kamiya et al., 2001; Takahashi et al., 2001). This work confirms Kölliker's organ contribution to tectorial membrane development providing new evidence of *Prdm16's* necessity for interdental cell development and tectorial membrane limbal domain formation. Additionally, this work shows that Kölliker's organ orchestrates spiral limbus development through a non-cell autonomous role.

Cochlear-specific deletion of *Prdm16* enabled the analysis of Kölliker's organ roles during cochlear postnatal development. Utilizing *Fgf20*-driven CRE recombination avoided *Prdm16* deletion from the developing nervous system and neural crest cells, thereby allowing the viability of animals for postnatal analysis (Gorski et al., 2002; Ohyama and Groves, 2004; Jacques et al., 2007; Zelarayan et al., 2007; Kang and Hébert, 2012). *Fgf20*-driven *Prdm16* deletion was efficient in the cochlear middle and apical turns, yet the basal turn partially escaped deletion possibly due to the onset of the *Fgf20*Cre recombinase activity in the base that is lagging *Prdm16* expression (Fig. 1*A–C*). Such finding limits our interpretation of basal cochlear phenotypes due to incomplete deletion yet allows for an intrinsic control region that can further validate the deletion effects observed in the middle and apical turns.

Deleting *Prdm16* using either a conventional mutant mouse model (*Prdm16^cGT^*; Ebeid et al., 2022) or an epithelial-specific conditional deletion model (*Prdm16* cKO used in this study) led to cochlear duct shortening that was accompanied with increased epithelial cell densities in the apical turn, confirming *Prdm16's* role in cochlear duct lengthening and excluding any *Prdm16* mesenchymal contribution in such a role (Fig. 2). Consistent with our previous findings, ectopic sensory islands were observed within the Kölliker's organ in *Prdm16* cKO, indicating a possible *Prdm16's* role in inhibiting prosensory genes within the Kölliker's organ domain during early development. These ectopic sensory patches were located within the medial part of the Kölliker's organ at the junction with Reissner's membrane which is the region of the developing interdental cells. We suggest this region has some capacity to generate hair cells during development. Gain-of-function experiment overexpressing *Prdm16* in developing cochlear cultures in vitro did not yield changes in hair cell density. Such observation indicates that *Prdm16*—as a sole factor—was not sufficient to inhibit sensory fates in the developing sensory epithelium.

Postnatal analysis of *Prdm16* cKO cochlear development showed defects in multiple structures, including the spiral limbus, inner sulcus, tectorial membrane, and interdental cells. Given the lack of interdental cell differentiation in *Prdm16* cKO cochlea, we believe that *Prdm16* functions as a permissive factor for interdental cell differentiation.

Lack of interdental cells was associated with the loss of the tectorial membrane limbal domain and, thereby, the loss of tectorial membrane anchorage to the spiral limbus. The tectorial membrane inconsistently maintained an attachment to the Reissner's membrane in *Prdm16* cKO (Figs. 5–7), simulating the phenotype of *Tecta^ΔENT/ΔENT^* mice lacking functional TECTA (Legan et al., 2000, 2014). Additionally, the tectorial membrane in *Prdm16* cKO showed significant volume loss as well as a decrease in COLLAGEN II and IX content, indicating a defect in collagen secretion by Kölliker's organ cells. TEM qualitative data shows that Kölliker's organ cells exhibit fewer microvilli in *Prdm16* cKO, which may contribute to the delayed collagen fiber secretion and organization seen in this model. As *Tecta* mRNA levels were not changed (as evident by *Tecta* mRNA FISH; Figs. 7, 12), we believe that delayed collagen secretion relative to TECTA protein secretion might contribute to the accumulation of glycoprotein aggregates under the covernet observed at P0.

In *Prdm16* cKO cochleae, the spiral limbus and inner sulcus failed to develop in the apical and middle turns, yet the basal turn showed no phenotype (Figs. 3, 4). This can be explained by the incomplete deletion of *Prdm16* in the basal turn of *Prdm16* cKO cochlea. This explanation is supported by our previous work using *Prdm16^cGT^* null mice (Ebeid et al., 2022) that showed the loss of spiral limbus and inner sulcus in all cochlear turns. In a recent work analyzing *Ebf1* role in cochlear development, inner ear epithelial-specific conditional *Ebf1* deletion led to lack of spiral limbus which was more prominent in the middle and apical turns. Since *Ebf1* is shown to be expressed in the Kölliker's organ (Kagoshima et al., 2024; Powers et al., 2024), such data align with our findings that Kölliker's organ orchestrate spiral limbus development.

**Figure 14.**
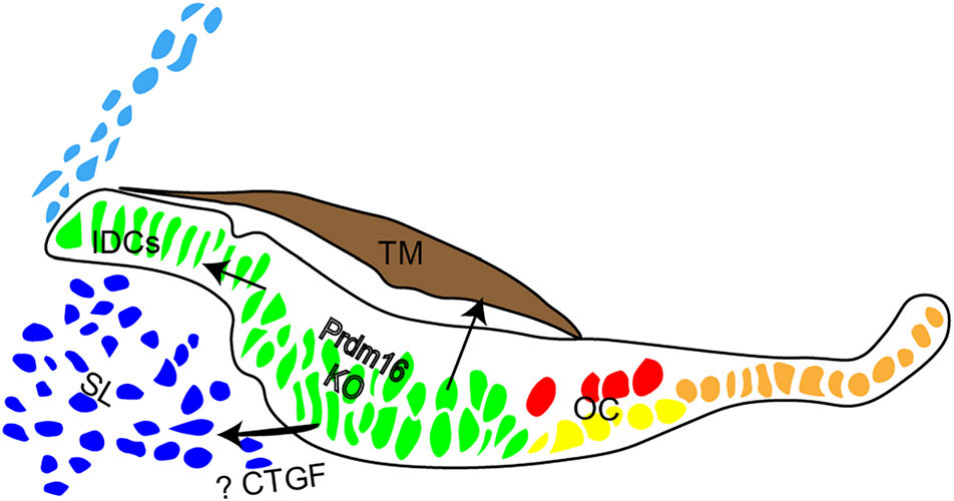
Schematic diagram of the developing cochlear duct illustrating proposed mechanisms of Kölliker's organ role in the development of different cochlear structures. IDCs, interdental cells; OC, organ of Corti; KO, Kölliker's organ; SL, spiral limbus; TM, tectorial membrane.

Mechanistically, we show that the spiral limbus in *Prdm16* cKO exhibited defective interstitial matrix deposition suggesting a non-cell autonomous role of Kölliker's organ epithelium in regulating underlying mesenchyme. To investigate epithelial-mesenchymal signaling mechanism involved in spiral limbus development, we conducted single-cell mRNA sequencing analysis followed by differential gene expression analysis within the Kölliker's organ of *Prdm16* cKO cochlea compared with control. We were able to identify a candidate gene coding for the secreted protein CTGF that was downregulated in *Prdm16* cKO Kölliker's organ. We validated the downregulation of *Ctgf* mRNA expression using FISH and real-time PCR within the middle and apical turns in *Prdm16* cKO Kölliker's organ. CTGF is an extracellular matrix protein that belongs to the cellular communication network (CCN) family of proteins (Bork, 1993) and plays a role in different cellular functions including proliferation, migration, differentiation, as well as synthesis of extracellular matrix (ECM) proteins in various cell types (Lau and Lam, 1999; Brigstock, 2003; Perbal, 2004). We hypothesize that CTGF protein is secreted from *Prdm16*-expressing Kölliker's organ cells and diffuse to the underlying mesenchyme to regulate matrix protein secretion and spiral limbus development (Fig. 14). An epithelial-specific *Ctgf* conditional deletion mouse model is currently being generated to test this hypothesis.

**movie 1.** 360° rotational video of the scala media from control cochlea. Amira 3D reconstruction of the scala media (green) from *Prdm16* heterozygote control cochlea at P28. [View online]

**movie 2.** 360° rotational video of the spiral limbus from control cochlea. Amira 3D reconstruction of the spiral limbus (blue) from *Prdm16* heterozygote control cochlea at P28. [View online]

**movie 3.** 360° rotational video of the tectorial membrane from control cochlea. Amira 3D reconstruction of the tectorial membrane (yellow) from *Prdm16* heterozygote control cochlea at P28. [View online]

**movie 4.** 360° rotational video of the spiral limbus and tectorial membrane (combined) from control cochlea. Amira 3D reconstruction of the spiral limbus (blue) and the tectorial membrane (yellow) from *Prdm16* heterozygote control cochlea at P28. [View online]

**movie 5.** 360° rotational video of the scala media from *Prdm16* cKO cochlea. Amira 3D reconstruction of the scala media (green) from *Prdm16* cKO cochlea at P28. [View online]

**movie 6.** 360° rotational video of the spiral limbus from *Prdm16* cKO cochlea. Amira 3D reconstruction of the spiral limbus (blue) from *Prdm16* cKO cochlea at P28. [View online]

**movie 7.** 360° rotational video of the tectorial membrane from *Prdm16* cKO cochlea. Amira 3D reconstruction of the tectorial membrane (yellow) from *Prdm16* cKO cochlea at P28. [View online]

**movie 8.** 360° rotational video of the spiral limbus and tectorial membrane (combined) from *Prdm16* cKO cochlea. Amira 3D reconstruction of the spiral limbus (blue) and the tectorial membrane (yellow) from *Prdm16* cKO cochlea at P28. [View online]

10.1523/JNEUROSCI.0721-24.2025.d1 Extended Data 1 **3D surface projection of the spiral limbus from control cochlea**. Amira 3D reconstruction of the cochlear spiral limbus from *Prdm16* heterozygote control cochlea at P28. Download Extended Data 1, FAS file.

10.1523/JNEUROSCI.0721-24.2025.d2 Extended Data 2 **3D surface projection of the tectorial membrane from control cochlea**. Amira 3D reconstruction of the cochlear tectorial membrane from *Prdm16* heterozygote control cochlea at P28. Download Extended Data 2, FAS file.

10.1523/JNEUROSCI.0721-24.2025.d3 Extended Data 3 **3D surface projection of the spiral limbus from *Prdm16* cKO cochlea**. Amira 3D reconstruction of the cochlear spiral limbus from *Prdm16* cKO cochlea at P28 showing lack of spiral limbus in the apical half of the cochlea. Download Multimedia/Extended Data, FAS file.

10.1523/JNEUROSCI.0721-24.2025.d4 Extended Data 4 **3D surface projection of the tectorial membrane from *Prdm16* cKO cochlea**. Amira 3D reconstruction of the cochlear tectorial membrane from *Prdm16* cKO cochlea at P28. Download Multimedia/Extended Data, FAS file.

Lastly, we identified hearing deficits in *Prdm16* cKO mice impacting most frequencies with the biggest threshold shifts at lower frequency ranges (4–8 kHz), possibly due to the severity of structural defects in the apical and middle cochlear turns. Since multiple structural defects are present in *Prdm16* cKO cochlea, it was not possible to attribute the functional loss to a specific structural defect. Nevertheless, we hypothesize that tectorial membrane phenotype is a major contributing factor to the hearing loss in these mice since similar tectorial membrane defects found in *Tecta* mutant mice have been linked to profound hearing deficits (Verhoeven et al., 1998; Xia et al., 2010; Legan et al., 2014). To determine if *Prdm16* haploinsufficiency contributes to hearing deficits in mice and to understand the pathophysiology of hearing loss secondary to *Prdm16* haploinsufficiency in 1p36 deletion syndrome (Heilstedt et al., 2003; Battaglia et al., 2008; Shimada et al., 2015; Jacquin et al., 2023), we compared *Prdm16* heterozygote hearing to littermate wild-type mice. Although *Prdm16* conditional heterozygote mice (*Fgf20^C/+^*; *Prdm16^fl/+^*) show statistically significant threshold shifts at 32 kHz at P60, we did not see any change in *Prdm16^cGT^* heterozygote mice at the same age, indicating the probability of *Fgf20* haploinsufficiency or background contribution to the observed deficit in heterozygote mice. We conclude that the loss of both copies of *Prdm16* is sufficient to cause hearing loss; however, the loss of one copy of *Prdm16* does not affect hearing in mice. Hearing loss in 1p36 deletion syndrome might be attributed to multiple gene haploinsufficiencies (including *Prdm16*) on the deleted segment of chromosome 1.

Based on such data, we propose that *Prdm16* expression within the Kölliker's organ and interdental cells is essential for cochlear development (Fig. 14) including the following roles: (1) *Prdm16* expression permits interdental cell development and thereby the formation of the tectorial membrane limbal domain and its proper anchorage, (2) *Prdm16* expression within the Kölliker's organ regulates the secretion of collagens essential for tectorial membrane matrix development, and (3) *Prdm16* regulates Kölliker's organ cells to signal the underlying mesenchyme possibly through CTGF to secrete extracellular matrix and develop the spiral limbus.

Kölliker's organ has been infrequently studied previously due to its transitional nature during development (Anniko, 1980; Uziel et al., 1981; Legrand et al., 1988; Tritsch et al., 2007; Dayaratne et al., 2014), yet we show in this study that it is instrumental to the development of multiple structures essential for hearing. This study establishes *Prdm16* as a novel gene associated with hearing loss in mice. We believe that *Prdm16* deletion mouse models have provided novel insight into Kölliker's organ's roles that set the stage for understanding the etiology of hearing impairment associated with *Prdm16* haploinsufficiency, as well as other possible genetic conditions involving dysregulation of Kölliker's organ development.
